# Impact of Plant-Based Meat Alternatives on the Gut Microbiota of Consumers: A Real-World Study

**DOI:** 10.3390/foods10092040

**Published:** 2021-08-30

**Authors:** Miguel A. Toribio-Mateas, Adri Bester, Natalia Klimenko

**Affiliations:** 1 School of Applied Sciences, London South Bank University, London SE1 0AA, UK; bestera@lsbu.ac.uk; 2 School of Health and Education, Middlesex University, London SE1 0AA, UK; 3 Center for Precision Genome Editing and Genetic Technologies for Biomedicine, Institute of Gene Biology, Russian Academy of Sciences, 119334 Moscow, Russia; lklimenko@genebiology.ru; 4 Research and Development Department, Knomics LLC, Skolkovo Innovation Center, 121205 Moscow, Russia

**Keywords:** gut microbiome, gut microbiota, plant-based meat alternatives, flexitarian, flexitarianism, plant-based diets, meat alternatives, meat substitutes, plant protein, ultra-processed foods

## Abstract

Eating less meat is increasingly seen as a healthier, more ethical option. This is leading to growing numbers of flexitarian consumers looking for plant-based meat alternatives (PBMAs) to replace at least some of the animal meat they consume. Popular PBMA products amongst flexitarians, including plant-based mince, burgers, sausages and meatballs, are often perceived as low-quality, ultra-processed foods. However, we argue that the mere industrial processing of ingredients of plant origin does not make a PBMA product ultra-processed by default. To test our hypothesis, we conducted a randomised controlled trial to assess the changes to the gut microbiota of a group of 20 participants who replaced several meat-containing meals per week with meals cooked with PBMA products and compared these changes to those experienced by a size-matched control. Stool samples were subjected to 16S rRNA sequencing. The resulting raw data was analysed in a compositionality-aware manner, using a range of innovative bioinformatic methods. Noteworthy changes included an increase in butyrate metabolising potential—chiefly in the 4-aminobutyrate/succinate and glutarate pathways—and in the joint abundance of butyrate-producing taxa in the intervention group compared to control. We also observed a decrease in the Tenericutes phylum in the intervention group and an increase in the control group. Based on our findings, we concluded that the occasional replacement of animal meat with PBMA products seen in flexitarian dietary patterns can promote positive changes in the gut microbiome of consumers.

## 1. Introduction

Demand for plant-based products is growing globally, led by increasing public knowledge of the extent to which our food choices affect human health. Researchers continue to unveil the strong links that exist between excessive meat consumption and the pathogenesis of non-communicable diseases such as obesity [1,2], type-2-diabetes [3,4,5], cardiovascular disease [6] and some forms of cancer [7,8]. Conversely, there is mounting evidence that diets characterised by a higher consumption of plant-based foods promote health and reduce the risk of a number of chronic conditions [9,10], and that high fibre and polyphenol content of plant-based diets promotes the changes in gut microbiota composition that mediate these positive health outcomes [11,12,13,14,15].

Recent research suggests that health- and environment-conscious flexitarians are more likely to incorporate animal-free foodstuffs—including plant-based meat alternatives—into their diets [16,17,18]. Research also shows that flexitarians, in addition to vegetarians and vegans, are more attracted to plant-based meat foods that imitate processed meat products, such as burgers, meatballs or sausages, than to those that imitate unprocessed meats, e.g., steak [19]. Additionally, dietary pattern analysis of consumers of plant-based foods has also found that convenience meals and snacks are highly appealing to this community [20]. These findings raise some concerns. Firstly, not all meat substitutes are sustainable. Some contain palm oil, known to increase pollution, GHG emissions and land conversion [21,22], and some may include genetically modified foods, which remain a contentious issue worldwide [23]. Secondly, certain meat substitutes may be classed as ultra-processed [24] because of their high fat or sodium content, or on the basis of a long list of what consumers consider “unnecessary ingredients”, i.e., preservatives, sweeteners, etc. [25,26,27].

The facts we have laid out above present us with an interesting paradox: although the evidence that plant-based diets promote human health continues to grow, the avoidance of animal-based foods has been found to be associated with a higher consumption of convenience, ultra-processed foods (UPFs) [28] that are typically seen as unhealthy. UPFs are known to include reduced amounts of ingredients of high nutritional value along with high levels of unhealthy fats and refined carbohydrates [29,30]. Fibre-poor UPFs are known to alter the provision of nutrient substrate to the colon due to differing digestibility, thereby promoting negative changes in both the composition and the metabolic activity of the gut microbiota [31,32], leading to a deranged state known as dysbiosis [33,34]. Some plant-based meat alternatives (PBMAs) may be classed as UPFs. However, we argue that the mere industrial processing of ingredients of plant origin does not make a PBMA product ultra-processed by default. In fact, we argue that the potential for a PBMA product to promote either eubiotic or dysbiotic changes in the gut microbiota of consumers lies in the nutrient profiles of each of its individual ingredients, and that quality assessments should be carried out on a product-by-product basis or, at the very least, on PBMA product lines made with highly similar ingredients by a manufacturer that follows the same food production techniques across its product portfolio.

There is a distinct lack of literature on the quality of PBMAs from rigorously designed intervention trials that helps compare the effects of PBMA products with those of conventional (animal) meat products on the microbiome of consumers. Therefore, our study aims to fill a knowledge gap in this intersection of nutrition, microbiology and consumer behaviour. We posit that PBMA products, such as burgers, sausages or meatballs, manufactured with all-natural plant-based ingredients that are rich in vegetable protein, fibre and phenolic compounds, can elicit positive changes in the gut microbiome of consumers when used to substitute their animal meat product equivalents, even if this substitution is only occasional, i.e., as in flexitarian dietary patterns. To test our hypothesis, we assessed the changes in the composition and functionality of the gut microbiota in a group of 40 participants, 20 of whom substituted the aforementioned animal products with their corresponding plant-based products for 4 weeks, compared to a size-matched omnivorous control group.

## 2. Materials and Methods

The materials and methods for this randomised controlled study are listed under the following sections.

### 2.1. Participants

#### 2.1.1. Recruitment Procedure

Prospective volunteers were sought by social media advertisement and 210 individuals expressed interest in participating. After reading the participant information sheet, 48 individuals responded via e-mail to say they were not eligible to participate due to either (1) failing to meet the inclusion criteria, or (2) meeting at least 1 of the exclusion criteria. The inclusion and exclusion criteria are listed below:

Inclusion criteria:

- No underlying health conditions requiring prescription medication.
- Aged between 18 and 55.
- A BMI between 18.5 and 29.9.
- No antibiotics in the past 6 months.
- No probiotic supplements in the past month.
- Eats red meat/poultry/fish/eggs/cheese daily.
- Does not eat plant-based meat substitutes.

Exclusion criteria:

- Immediate DNA family (mother, father, brother, sister) with a medical diagnosis of ulcerative colitis, Crohn’s disease, irritable bowel syndrome, or bowel cancer.
- Allergic to soya.
- History of mental health disorders or brain cancer.
- Diagnosed with a condition for which they receive NHS support.
- Positive COVID-19 diagnosis or suspected COVID-19 symptoms in the previous 6 months.

Forty-two volunteers returned a signed consent form.

**Figure 1:**
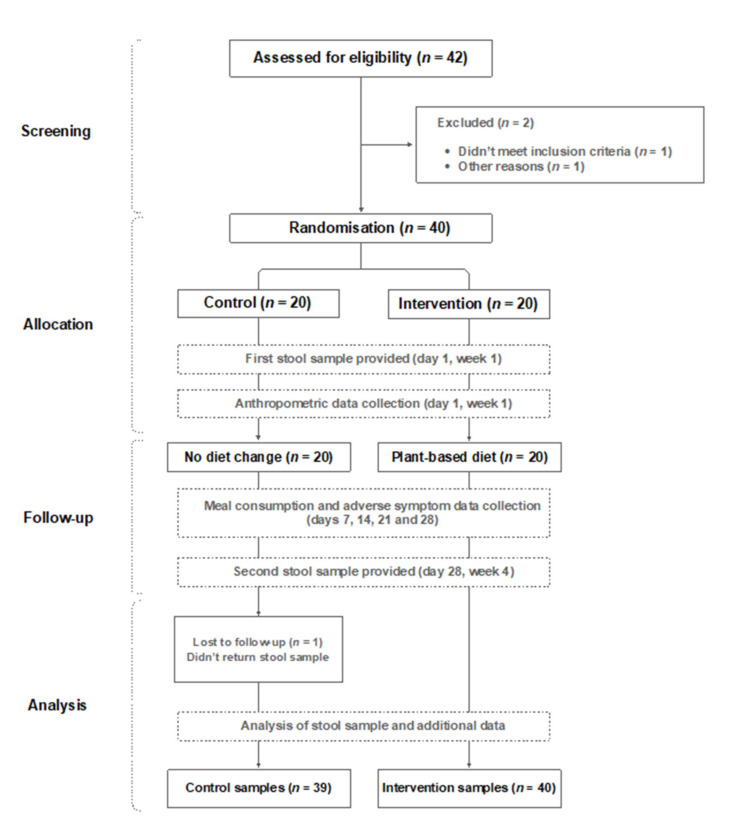
Study flow diagram.

Two participants withdrew before the study commenced. One was unable to return to the UK due to COVID-19 lockdown, and another had just started a course of antibiotics. An additional participant was withdrawn due to not returning the first stool sample. Thirty-nine healthy volunteers (19 male, 20 female) completed the entire 4-week study period (Figure 1). Baseline characteristics (mean ± SD) of the 39 individuals who completed the study were: age 37.5 ± 8.9 years (range 21–55); body weight 70.3 ± 11 kg (range 55–90); BMI 23 ± 2.3 (range 19–27).

The study was fully explained to the volunteers, in writing, and each gave their written, informed consent before participating. The School of Applied Science Ethics Committee, London South Bank University, approved the study. The ethical approval reference number is ETH2021-0025.

Data collection for the study took place during January and February 2021.

#### 2.1.2. Randomisation and Group-Allocation Procedure

The Sealed Envelope Ltd software [35] was used to apply the stratified block randomisation method to randomise participants into 2 groups (A, B), stratified for gender. One person not directly involved in the study chose the group identity, assigning A to the intervention group and B to the control group.

#### 2.1.3. Plant-Based Products Consumed by the Intervention Group

Participants consumed a selection of commercially available plant-based protein meat substitute products donated by The Meatless Farm, Leeds, UK [36]. They received a selection of plant-based mince, plant-based burger patties, plant-based sausages, plant-based sausage patties, and plant-based meatballs, commercially available in the United Kingdom, the United States of America, Canada, and the European Union. This is noteworthy in that the intervention products are real-world products and not products manufactured solely for the purpose of the study. The meatballs and patties are foodservice products at the time of writing this paper. The nutritional composition of the intervention products is presented in Table 1.

Full product information sheets can be found in the Supplementary Materials section for a complete nutrient breakdown, in addition to food safety considerations, storage, and cooking instructions.

Pea was the main source of protein in all products, with the exception of the plant-based mince. Phytonutrient analysis of pea flour, pea protein concentrate (dry fractionated) and dehulled peas is provided in Table 2. We should like to highlight at this point the levels of phenolic compounds such as lutein, ferulic acid and genistein, the relevance of which will be explored in the results discussion.

#### 2.1.4. Study Design and Procedure

The study adopted a randomised controlled, pre-and post-intervention assessment design to investigate the effects of regular consumption of plant-based foods on the gut microbiota of participants in comparison to a control group. Participants in the control group received no intervention. Instead, they were requested to carry on consuming animal products including red meat, poultry, fish, eggs, and cheese daily, i.e., they simply continued to adhere to the kin d of dietary pattern that was specified in the inclusion criteria, as per Table 1 above. All researchers, with the exception of the person who managed the participants, were blinded to the group allocation of the participants until the data analysis was concluded.

Both groups of participants received two stool test kits (Atlas Biomed, UK). Once they returned their first completed stool sample, the intervention group received the first delivery of plant-based ingredients (including mince, meatballs, sausages, sausage patties and burgers) sufficient to cook 1 plant-based meal per day for 14 days. Participants were requested to replace a minimum of 4 animal-protein based meals with a plant-based meal per week for four weeks and to log the number of meals at which they consumed the meatless products they were supplied with. At the end of the first two weeks, the plant-based ingredient delivery was repeated.

For the convenience of participants, and to optimise compliance with the study protocol, enough plant-based ingredients were sent to feed all members of the household. The ingredients were sent frozen with thawing instructions, along with a recipe booklet provided by the organisers with ideas (26 in total) for participants to cook the PBMA products provided for the intervention. The recipe booklet sent to participants can be found in the Supplementary Materials section.

At the end of the 4-week period, participants in both the intervention and the control group were reminded to complete the second stool test and to send it to the laboratory.

#### 2.1.5. Participant Data Collection

After enrolment and randomisation to group allocation, participants were asked to provide anthropometric data. Two tables (one detailed and one summarised) are available from the Supplementary Materials section. Unless otherwise stated, data are expressed as mean ± SD. Anthropometric characteristics were compared using the one-way ANOVA for numerical values, and Fisher’s Exact Test for nominal values. Data were analysed using IBM SPSS (IBM, Armonk, New York, NY, USA) (Statistics for Windows, Version 25.0) [37].

At this point, participants received two gut microbiota test kits (Atlas Biomed, UK [38]) with detailed instructions on how to collect the samples in the convenience of their own home. The first stool sample was collected and returned by all participants before the intervention started, and the other stool sample was collected and returned by all participants at the end of the 4-week intervention period (on day 28). Participants in the intervention group provided consumption data and side effect/adverse symptom data at the end of each of the 4 weeks to the researcher managing the interventions and participants. A flow diagram of the study can be seen in Figure 1. Comprehensive anthropometrics, meal consumption data and self-reported side effects per week are available in separate files in the Supplementary Materials section.

### 2.2. Gut Microbiome Analysis

#### 2.2.1. Sample Collection

Microbiome sample collection was performed using the Omnigene Gut OM-200 kit [39,40], provided by UK-based biotech company Atlas Biomed [41] in ISO 13485:2016-accredited packaging (medical device) [42]. The OM-200 kit facilitated the DNA extraction and stabilisation [43] prior to being sequenced by the Illumina MiSeq platform [44,45]. The raw data were then analysed using the Deblur algorithm [46]. Variable trimming length was used: the maximum value (max_len) was determined as the most frequent read length across all samples, and then each value of trimming length in the range [max_len-1:max_len-4] was processed separately. The taxonomic classification of the denoised reads was performed with the QIIME2 Naive-Bayes classifier [47]. The classifier was trained on the Greengenes database v.13.5 [48] with 97% OTU similarity. ASVs (amplicon sequencing variants) detected in negative control samples that were known to be skin or environmental dwellers were removed from the analysis. For alpha-and beta-diversity analysis, the classified reads were randomly rarefied to the same number (3000 reads per sample), for each sample. Estimation of alpha-diversity for each sample was performed using the Shannon [49] and Chao1 diversity metrics [50]. Beta-diversity (pairwise dissimilarity between the gut community structures) was estimated using a Bray–Curtis dissimilarity metric [51,52]. Read counts of microbial species, genera, and families were calculated as the sum of reads assigned to the ASVs (amplicon sequencing variants) belonging to the respective taxon.

#### 2.2.2. Extended Statistical Analysis of the Microbiome Data

There is increasing awareness of the fact that microbiome datasets generated by high-throughput sequencing of 16S rRNA gene amplimers are compositional because they have an arbitrary total imposed by the instrument. For this reason, we performed the microbiome data analysis in a compositionality-aware manner, replacing zero values in the abundance table after removing all taxa with relative abundance greater than 1% in fewer than 5 samples. For the remaining taxa, all zero observations were replaced using a Bayesian-multiplicative replacement method (the CMultRepl function from the zCompositions package by Palarea-Albaladejo and Martín-Fernandez [53].

Our compositionality-aware approach to analyse beta-diversity included evaluation of Aitchison distance—the Euclidean distance in *clr* coordinates—with further Principal Coordinate Analysis (PCoA). Significant associations with the time-point were identified via the PERMANOVA test for each group separately. Associations of beta-diversity between paired samples (magnitude of change) and metadata (age, gender, BMI, weight and number of consumed meals) were analysed using a linear model for each group separately. Associations of beta-diversity between the paired samples with intervention group were also analysed using a linear model. 

Associations of alpha-diversity changes (Shannon and Chao1 indexes) with intervention group were analysed using ANCOVA analysis with the following formula:


*(diversity after intervention) ~ intercept + (diversity before intervention) + group*

Associations of alpha diversity change with metadata were calculated using linear regression separately for each of the groups.

The taxonomic differential abundance analysis in a compositionality-aware manner was implemented using two different approaches: within-group and between-group analyses. Firstly, the differences in taxa balances were estimated in each group separately with the DBA algorithm (discriminative balance analysis) [54]. The DBA variant which favours smaller balances was selected (*sbp.fromADBA*). The algorithm selects *ilr* (isometric log ratio) coordinates based on the metadata (in our case, the time point). Then we calculated balance values in selected coordinates. Next, using a linear regression (mixed-effect model with subject ID as a random effect) we evaluated the significance of change for each balance. We then performed multiple comparison correction using the Benjamini–Hochberg method to control the False Discovery Rate (FDR) [55,56].

Secondly, between-class analysis was performed to investigate if the changes of taxa abundances differed between groups. In order to eliminate the changes which appeared because of baseline differences between the groups, we included an additional filtration step involving the removal of taxa for which the difference between groups before the intervention was higher than the difference after the intervention. In summary, the protocol for our analysis procedure included the following steps:

- Detection of the taxa for which the between-group differences before the intervention were lower than between-group differences after the intervention (using ALDEx2 algorithm [57]);
- Calculation of the matrix of bacterial changes (abundance_after/abundance_before) using only bacteria detected by ALDEx2;
- DBA on matrix of bacteria changes using interventional group as a factor (sbp.fromADBA) to obtain *ilr* coordinates;
- Calculation of the balance values before and after the intervention using DBA-defined *ilr* coordinates;
- ANCOVA analysis for each balance using the following formula:
  *(balance after intervention) ~ intercept + (balance before intervention) + group*
- Estimation of the *p*-values for group factor and FDR correction.

#### 2.2.3. Gut Microbiome Metabolic Potential Estimation

To estimate the metabolic potential of the microbial community we conducted closed-reference OTU picking using the usearch algorithm implemented in QIIME1.9 [58] against a 16S rRNA sequence database (Greengenes v. 13.5 [48], 97% OTU similarity) followed by version 1 of the Phylogenetic Investigation of Communities by Reconstruction of Unobserved States (PICRUSt1) algorithm [59]. Butyrate-producing activity was estimated using the Knomics-Biota bioinformatics platform [60], where curated butyrate-producing pathways abundances were estimated. We analysed differences in butyrate pathway abundance changes between groups using ANCOVA with further Benjamini–Hochberg (FDR) correction for enhanced statistical precision [55].

In addition, we estimated the change of butyrate-producing potential based on the abundance change of its producers. For this purpose, we created a list of butyrate-producing taxa from the literature and ensured that these microbes had non-zero abundance in our data. We used these taxa as the numerator in the balance, and all other microbes were used as denominators. The balance was checked for association with a group using ANCOVA analysis.

## 3. Results

### 3.1. Participant Data: Meal Consumption and Side Effects

Participants in the intervention group (group A) were asked to report the number of meals cooked with the plant-based foods provided consumed per week. The averages (mean ± SD) were 5.97 ± 2.70 for week 1, 5.39 ± 1.70 for week 2, 5.10 ± 1.81 for week 3 and 4.53 ± 1.23 for week 4. This resulted in an average across the 4 weeks of the study of 5.20 ± 0.56. Taking into consideration that each of the 26 recipes in the booklet contained a minimum serving per person of 100 g of the PBMA products provided, and that the average fibre content of these PBMAs (as per Table 1 above) was 3.65 ± 0.56 (mean ± SD, in grams per 100 g), that means that every participant in group A added an average of 18.98 g of fibre to their weekly diet during the study. This might explain why >50% of group A participants reported increased gas/bloating at each of the three follow-ups, making this the most commonly reported adverse symptom. Consumption of a high fibre diet is known to delay intestinal gas transit by driving changes in the composition of the gut microbiota, in addition to decreasing bolus propulsion to the rectum, which may elicit gaseous symptoms by promoting gas retention [61]. However, we found no statistically significant association between increased intestinal gas and number of meals consumed per week (Pearson chi-square *p* > 0.1), with only a weak indication (Pearson chi-square *p* > 0.06) of such possibility at the first follow-up (day 7). We also did not find a statistically significant association between increased gas and gender (Fisher’s exact *p* > 0.1).

Other side effects reported by some of the participants included “less lethargic, constipation has improved” (*n* = 1), “improved stool consistency” (*n* = 1), “more regular bowel movements (*n* = 3), “bowel movements have increased in volume by 100%” (*n* = 1), “bowel movements more regular and tiredness after meals have disappeared” (*n* = 1), “improvement of IBS symptoms” (*n* = 1), and “stomach discomfort several hours after consumption” (*n* = 1). Given the small sample size and the heterogeneity of the comments, it was not deemed necessary to perform a thematic analysis of this qualitative data.

All participant comments were recorded in a spreadsheet entitled “Meal Consumption and Adverse Effects”, which is available in the Supplementary Materials section. This document also contains the statistical analyses mentioned previously in this section.

### 3.2. Baseline Taxonomic Composition of the Participants’ Microbiota

Baseline microbiome composition of the participants stool was dominated by Bacteroides (23 ± 13%), Prevotella (10 ± 17%) and Faecallibacterium (9 ± 4%) (Supplementary Figure S1). In general, microbiome composition corresponded to the previously described stool microbiome content of the urban population [62]. The diversity (Shannon and Chao1 metrics) did not differ between the intervention and the control groups at baseline (Student’s t test, *p* > 0.8). Similarly, there were no differences in taxa balances (defined by DBA, see Methods) between groups (linear model FDR > 0.2).

All samples except one (099-756-826, Control group) showed a good quality expressed in a high number of mapped reads (38,734 ± 4387). Sample 099-756-826 with low coverage (seven reads mapped) and its paired sample were excluded from the analysis.

### 3.3. Microbiome Composition Changes Due to the Intervention

#### 3.3.1. Beta-Diversity

**Figure 2:**
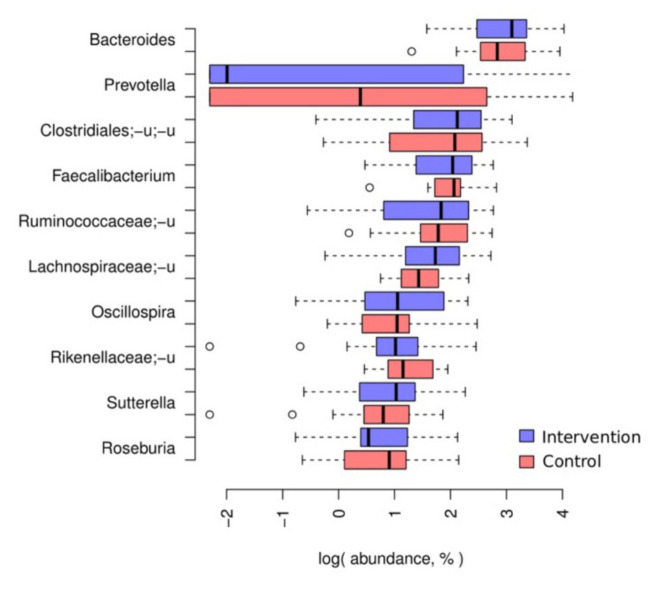
Major taxa. Boxplots detailing the distribution of relative abundance for the 10 most abundant genera; for appropriate display on a log scale, zero values were replaced with a pseudocount (0.1%). In addition, boxplots detailing the distribution of relative abundance for the 25 most abundant taxa in each taxonomic rank at baseline are available in the Supplementary Materials section.

Overall microbiome composition change measured as beta-diversity between paired samples before and after the intervention (Aitchison distance) was not significantly associated with the study group (linear model, *p* = 0.3, Figure 2). However, when within-group changes were estimated (PERMANOVA analysis with Stata 17 [63]), a significant result was detected only in the intervention group (paired distance 6.46 ± 2.46), but not in the control group (paired distance 5.78 ± 1.20). We also compared the magnitude of change with all participant metadata, including age, gender, BMI, and weight, separately in each group, and with the number of meals consumed by participants in the intervention group. No significant results were found in this analysis (*p* > 0.1).

#### 3.3.2. Between-Group Differential Abundance Analysis

**Figure 3:**
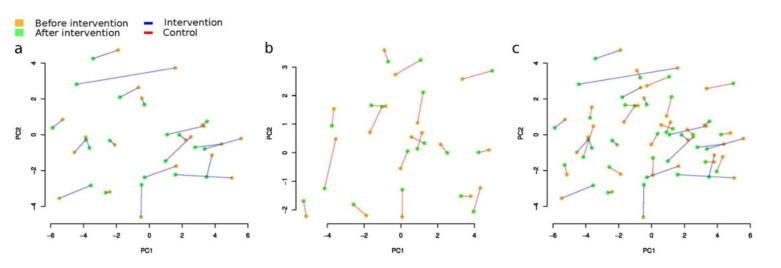
General changes of microbiome composition after the intervention (Aitchison distance): (**a**) intervention (Group A) (PERMANOVA *p* = 0.01, R^2^ = 0.88%); (**b**) control (Group B), (*p* = 0.571, R^2^ = 0.27%); (**c**) intervention and control groups combined. Please note that the red dots denote the samples before the intervention, and the blue dots, after.

As a result of between-class differential abundance analysis, we obtained a balance (DBA, please see Methods) associated with the difference of microbiome changes in group A (intervention) and group B (control). The outcome of this biostatistical analysis revealed the relationship between *Coprococcus* and *Roseburia* and between Parabacteroides and unclassified genera from the Tenericutes order ML615J-28 (ANCOVA, FDR > 0.05, Figure 3). The first balance tended to decrease more in the intervention group than in the control, whereas the second shows precisely the opposite association.

#### 3.3.3. Within-Group Differential Abundance Analysis

**Figure 4:**
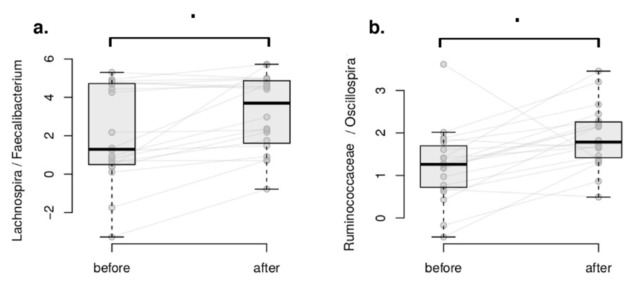
Within-group differential abundance analysis using DBA. Microbial balances associated with the plant-based intervention. (**a**) *Lachnospira*/*Faecalibacterium* balance. (**b**) *Ruminococcaceae*/*Oscillospira* balance. Dots denote associations very close to significance (FDR < 0.06).

**Figure 5:**
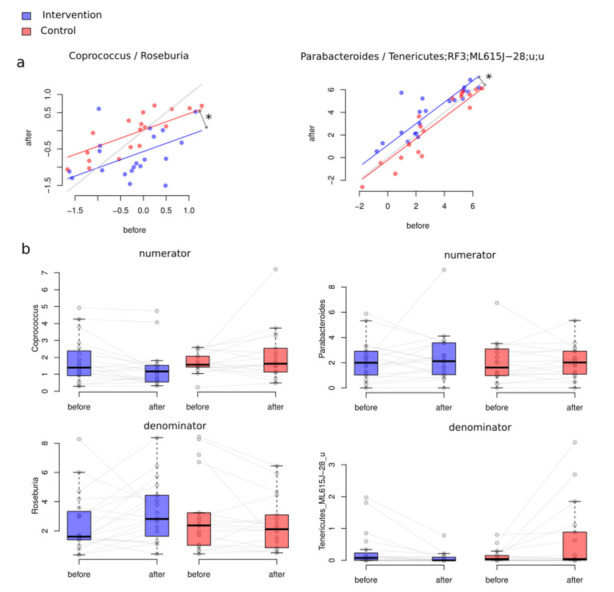
(**a**) Between-group differential abundance analysis results. Significantly changed balance values before and after the intervention in both the control and the intervention groups. Figure interpretation is as follows: There is one point per participant. If the point is under the grey line, for the participant, the balance decreased. If the point is above the line, the balance increased. Asterisks denote significant associations (FDR < 0.05). (**b**) The relative abundance of the taxa included in numerators and denominators of significantly changed balances (%). If we consider these relative abundances, it can be suggested that the main impact in the second balance was provided by unclassified Tenericutes.

The differential abundance analysis of taxa aims to detect differences in taxonomic composition between samples or conditions [64,65,66]. Here, differential abundance analysis was applied to microbial balances to investigate the microbiome composition changes in each of study groups following a compositionality-aware method [54,64,65,66]. Microbial balance is a ratio between two groups of bacterial taxa (numerator and denominator) calculated in a specific manner and used as a microbiome composition feature; for details, please refer to the protocol by Egozcue et al. [67]. No statistically significant changes of taxa balances were found in either group (FDR > 0.05), as defined by DBA. Please refer to the Methods section for DBA definition. However, we identified a noteworthy, marginally significant trend (0.05 < FDR < 0.06) in the balances of the *Lachnospira*/*Faecalibacterium* and *Ruminococcaceae*/*Oscillospira* taxa in the intervention group only, whereas no such behaviour was seen in the control group, as seen in Figure 4 and Figure 5.

In the balances concept, the numerator of the balances (*Lachnospira* and *Ruminococcaceae* unclassified) increased in relation to the denominator taxa (*Faecalibacterium* and *Oscillospira*, respectively) in the intervention group. Interestingly, both numerator and denominator members include butyrate producers [68,69,70,71,72] and taxa associated with healthy dietary patterns.

Interactive heatmaps representing the relative abundance of major microbial genera in the samples at day 1 and day 28 of the study for the intervention and control groups are available at: http://bit.ly/ETH2021-0025a.

Alternatively, the analysis was replicated with the selbal method. The method revealed one significant association (unclassified Tenericutes_ML615J-28)/*Sutterella* balance (cross-validation AUC = 0.68 ± 0.11). In this balance only unclassified Tenericutes showed reliable reproducibility (74%), whereas *Sutterella* can only be considered an unreliable part of the balance due to its low reproducibility (34%).

Summarising the results of the two analyses, we can conclude that the change in Tenericutes_ML615J-28 abundance was significantly different in the intervention group compared to the control; namely, it relatively decreased in the intervention and relatively increased in the control group.

#### 3.3.4. Alpha-Diversity Changes

**Figure 6:**
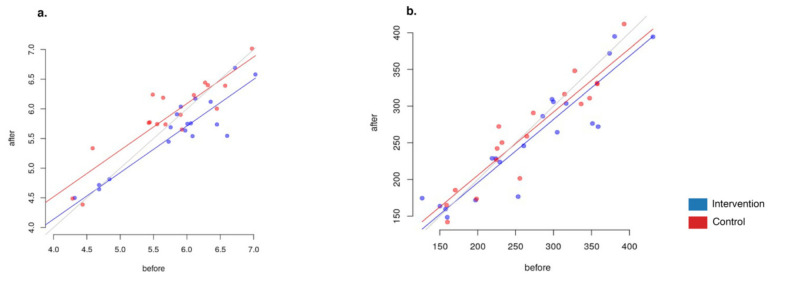
Alpha-diversity values before and after the intervention. (**a**) Shannon index ANCOVA (*p* = 0.004). (**b**) Chao index ANCOVA (*p* = 0.243).

Overall, the Shannon index decreased slightly in the intervention group (group A), with a Shannon index change from 5.65 ± 0.94 to 5.44 ± 0.85 (Welch’s test *p* = 0.45 × 10^−5^, *n* = 40 samples), and increased in the control group (group B), with a Shannon index change from 5.65 ± 0.94 to 5.44 ± 0.85 (Welch’s test *p* = 0.57 × 10^−5^, *n* = 38 samples). The changes were significantly different between the two groups (ANCOVA *p* = 0.004, Figure 6). This change was not associated with age, gender, BMI or weight in either group (*p* > 0.05). Interestingly, the fractional decrease in diversity in the intervention group was not associated with the reported weekly number of meals cooked with the plant-based meal alternative (PBMA) ingredients provided (*p* > 0.1).

We found no significant differences in diversity by Chao1 index between the groups (ANCOVA *p* = 0.243)

### 3.4. Potential Changes to Butyrate-Producing Taxa

We estimated the butyrate-production potential change in three alternative ways. Firstly, we calculated the balance by placing the main taxa involved in the production of butyrate in human gut in the numerator, and all other taxa in the denominator. The following butyrate producers were assessed: *Faecalibacterium*, *Eubacterium*, *Roseburia*, *Ruminococcus*, and *Anaerostipes*.

**Figure 7:**
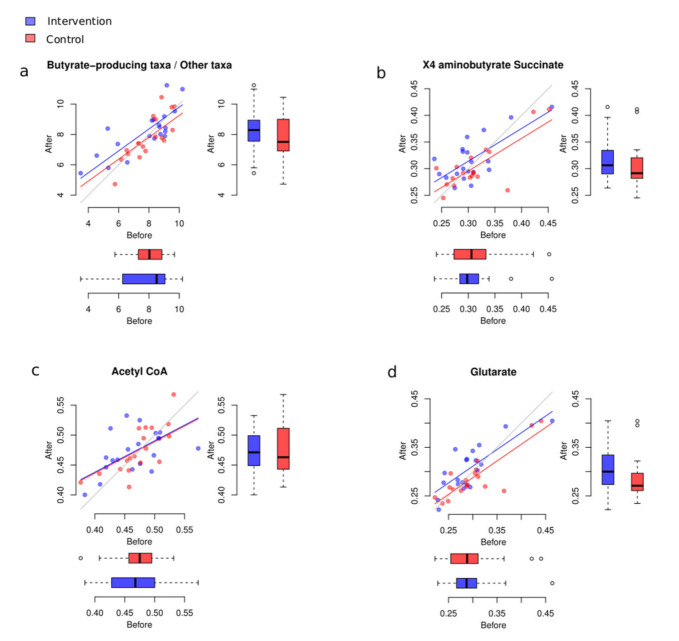
Changes to taxa with butyrate-production potential. (**a**) Changes of balance between main butyrate-producing taxa and all other bacteria in the intervention and control groups. (**b**–**d**) Butyrate synthesis pathways before and after the intervention in two groups.

The change of this balance was not significantly different between groups (*p* = 0.08). However, we observed a slightly stronger increase in participants who consumed the plant-based foods provided for the intervention, compared to those in the control group (Figure 7).

Secondly, we investigated the *Lactobacillus* and *Bifidobacterium* genera for their reported cooperating relationship with butyrate producers [73,74,75]. We found their total change was not significantly different between the groups (*p* = 0.14). 

Thirdly, we analysed the relative abundance of three curated pathway variants for butyrate synthesis commonly present in human gut microbes, using a biostatistical method covered in the Supplementary Materials section. Changes in the “glutarate” pathway were significantly different between the two groups (FDR = 0.0382, Figure 7b–d). For the “4-aminobutyrate/succinate” pathway, we observed a non-significant trend in the same direction (FDR = 0.0891).

### 3.5. Availability of Data for Educational and Research Purposes

Being educators in the field of microbiome and food science, we are acutely aware of the need for real-world datasets that can be used for educational purposes. For that reason, we are pleased to make all FASTQ available on open access for educational use. They can be downloaded from: dataview.ncbi.nlm.nih.gov/object/PRJNA738373?reviewer=5h6j0rs8dejqdch5ts6ur5n0t7. The project id is PRJNA738373.

## 4. Discussion

### 4.1. Review of Findings

One of the greatest challenges in defining “a healthy gut” is that most of the variance (~85%) within the human microbiome is still unaccounted for, as confirmed by population-wide studies [76,77]. However, the relative abundance of microbes able to ferment non-digestible substrates such as dietary fibres to produce short-chain fatty acid (SCFAs) continues to be one of the indisputable criteria for such a definition [78,79,80,81,82]. Of the major SCFAs produced—acetate, propionate and butyrate—butyrate is particularly important for the simple reason that it constitutes the main energy source for colonocytes [83]. Butyrate can also activate intestinal gluconeogenesis, having beneficial effects on host glucose and energy homeostasis [84]. In addition, depletion of this microbe-derived metabolite is linked to several non-communicable diseases, such as type 2 diabetes (T2D) [85], obesity [86] and cardiovascular disease [87]. Furthermore, a reduced abundance of butyrate-producing genera has been shown to facilitate the establishment of enteric pathogens in animal models [88,89], and it has been associated with markers of systemic inflammation, e.g., C-reactive protein (CRP) in people living with inflammatory bowel diseases such as Crohn’s [90] and ulcerative colitis (UC) [91]. Although we did not observe a statistically significant increase in the relative abundance of any particular butyrate-producing taxon, we did identify a significant increase in the butyrate-production pathways only in the intervention group, with the glutarate butyrate-metabolising pathway being of particular significance. Moreover, trends in the same direction were detected for the acetyl-CoA and X4-aminobutyrate-succinate pathways, and in the joint abundance of butyrate producers. Amongst these microbes, we identified a noteworthy, marginally significant trend in the balances of the *Lachnospira*/*Faecalibacterium* and *Ruminococcaceae*/*Oscillospira* taxa in the intervention group only, whereas no such behaviour was seen in the control group. Genera within the *Ruminococcaceae* and *Lachnospiraceae* families are well documented as butyrate producers [68,69,92,93,94]. In addition, metagenomic screening of 3184 sequenced bacterial genomes from the Integrated Microbial Genome database by Vital et al. [95] suggests that genera in the *Ruminococcaceae* and *Lachnospiraceae* families are representative of the butyrate-producing taxonomic core characteristic of individuals with healthy colons. Furthermore, members of the *Oscillospira* genus, such as *Flavonifractor plautii*, have been found to be more abundant in individuals who adhere to dietary patterns characterised by the abundance and variety of fibre and polyphenol sources [60,71,96,97]. *Faecalibacterium* species, such as *Faecalibacterium prausnitzii*, have also been documented to thrive on both Mediterranean-type and plant-based dietary patterns [98,99,100]. The PBMA products provided contained a range of soluble and insoluble fibres from chicory root, carrot, pea, and potato (please see the Supplementary Materials section for full details) including hemicellulose, pectin, lignin and cellulose, which have been found to have a range of benefits to human health [101]. In addition, the phytonutrients in whole pea, pea flour and pea protein concentrate—the base ingredients of the PBMAs consumed by the intervention group—also contained phytonutrients such as lutein, genistein, daidzein and ferulic acid, all of which have been reported as modulating agents of the gut microbiome, favouring the growth of SCFA-metabolising microbes [102,103,104,105]. Butyrate plays a critical role in health and disease [95]; therefore we cautiously interpret the joint abundances of butyrate-producing microbes and the heightened presence of synthesis pathways in the intervention group as a positive sign of the effects of the substitution of meat-containing meals with PBMA-containing meals. A study with a larger number of participants should enable us to confirm our interpretation. Additionally, we interpret the observed changes in butyrate-producing bacteria and pathways as consistent with the microbial signature of “a healthy gut”, and thus see them as a confirmation of our hypothesis that not all PBMAs are necessarily ultra-processed and damaging to the human gut microbiome.

It is important to mention that the replacement of animal meat with the PBMA products provided was encouraged but not mandatory. Our aim was to understand the potential impact of the PBMAs on the gut microbiome in real world settings, in which consumers are free to eat as many or as few PBMA-containing meals per week as they wish. Moreover, the recipes in the booklet provided to participants in the intervention group also made it explicit that the substitution of meat with one of the PBMAs was to be carried out as part of a healthy balanced diet. For clarity, the intention was not for participants to consume PBMAs exclusively, but to use these products in cooking as part of their existing dietary pattern. We did account for the number of PBMA-containing meals consumed by participant per week and found that an average of 5 was sufficient to elicit the positive changes we observed.

We were particularly interested in the change experienced by the Tenericutes phylum. First and foremost, overall Tenericutes levels decreased in the intervention group and increased in controls, and the statistical significance of the difference in relative abundance between groups was strong. This behaviour was observed in the landmark study by David et al., in which researchers demonstrated how short-term consumption of diets composed entirely of animal or plant products alters microbial community structure. In this study, the Tenericutes phylum increased in participants who consumed mostly animal products, compared to those who consumed mostly plants [106]. Secondly, the most prevalent Tenericutes taxon in our sample, namely the ML615J-28 order, has not been previously associated with diet change or phenotypic change in humans, and the little observational evidence that is available for this taxon in diseased human cohorts does not help explain this change. A study by Bonder et al. [107] assessed the modulation of the gut microbiomes of 21 healthy volunteers who followed a gluten-free diet for four weeks. The authors found the Tenericutes ML615J-28 order decreased as a result of the exclusion of gluten. This is interesting because the plant-based products provided for the intervention group were gluten free, but participants were not advised to reduce or indeed to exclude gluten from their diets for the duration of the study. Although data about gluten consumption was not collected, we are not aware—albeit informally—of such a behaviour in the intervention group. Although the PBMA products provided to the intervention group were indeed gluten free, participants were not encouraged to exclude gluten-containing foods from their diet during the study. Hence, we find the similarity of our findings and those of Bonder et al. coincidental. Another study by Patrone et al. [108] found that Tenericutes tended to increase in mice fed a diet high in polyunsaturated fat compared to those fed a diet high in medium chain triglycerides from coconut. The plant-based products provided to the intervention group did contain small amounts of coconut oil. However, given the lack of substantial clinical precedent for the dietary modulation of Tenericutes, these arising hypotheses would need further testing in order to be confirmed. One murine model study found Tenericutes levels decreased significantly in a group of mice fed oolong tea extract and citrus peel—both rich sources of phenolic compounds—in addition to the amino acid L-carnitine, compared to mice that were fed the L-carnitine alone [109]. L-carnitine is one of the most prevalent amino acids in meat. It is metabolised by intestinal bacteria to trimethylamine N-oxide (TMAO), a compound that has been associated with cardiovascular disease [109] and some types of cancer [110,111]. Chen and colleagues measured the levels of the pro-inflammatory markers in both mouse groups and found that TMAO levels were greatly reduced in mice that were fed the tea and citrus extracts compared to controls, suggesting that dietary phenolic compounds in oolong tea extract and citrus peel reduced TMAO formation ability by gut microbiota, downregulating carnitine-induced vascular inflammation [109].

We also observed small changes in both alpha- and beta-diversity in both groups. The intervention group experienced a small decrease in alpha-diversity, whereas the control group experienced a small increase in alpha-diversity that did not bear a statistically significant association with the number of PBMA meals consumed. As per the study limitations (please see below), our statistical analysis did not take into account the putative effect of the increased fibre and phenolic compounds in the fresh fruit, vegetables, pulses, whole grains, etc. consumed with the PBMA foods provided. This, however, may be a moot point, on the basis that the PBMAs provided to the intervention group were meant to be consumed as part of a dietary pattern that features sources of dietary fibre and polyphenols. The main limitation of the study was our sample size. Based on that factor alone, it is difficult to establish the relevance of these changes. Interestingly, a slight but significant decrease in alpha-diversity was observed after a short-term high-fibre dietary intervention involving 16S rRNA sequencing of stool samples from 248 citizen-science volunteers. Although the effect appeared to be opposite to the correlation between diversity and long-term vegetable consumption clearly seen in that cohort, there is evidence to suggest that alpha-diversity may be sensitive to short-term dietary changes, but that longer intervention times are needed in order to assess the permanence of such changes [62]. The authors interpreted these unexpected changes as “shock effects of a relatively rapid change in the spectrum of incoming nutrients, which may transiently disrupt the ecology of the gut community”, or as a facet of microbiota “stress” linked to this transitory period [62]. The importance of allowing enough time for the response to dietary interventions to be considered to be stable/permanent has been documented in several high-impact publications. In addition, we agree with Johnson and Burnet of Oxford University that lessons from wider ecological systems demonstrate that diversity is just one of myriad factors to consider when analysing an ecosystem, and that its stability, structure and function are equally important [112]. Therefore, we see the marginal changes in alpha- and beta-diversity as part of the natural state of self-organisation of the gut microbiome, which we see as a living complex adaptive system engaging in ongoing acclimatisation, one of the principal characteristics of complex-adaptive dynamics [113,114]. The gut microbiome is constantly being challenged by ongoing micro-perturbations, e.g., ingestion of different compounds from different plant-based foods, each day; thus, it is safe to say that it is in a constant state of flux. In a recent study based on daily sampling of 34 healthy participants over 17 days, Johnson et al. [115] demonstrated that microbiome composition can be altered in as little as 48 h, and that daily microbial responses to diet were highly personalised. On that basis, including data from interim microbiome tests, e.g., asking participants to provide one sample every 7 days, may have added to the robustness of the study by providing better insight into the stability of the observed changes.

### 4.2. An Interesting Paradox

Despite the undisputed health benefits of fruit and vegetables, nuts and seeds, whole grains and legumes [116,117,118,119,120], the reality is that meat and other animal products continue to be the central element of many dietary patterns and cultures around the world [121]. As an example, recent cross-sectional analysis using data from the UK National Diet and Nutrition Survey published by researchers at the University of Reading found that 43% of British adults (men 57% and women 31%) consumed more than the 70 g/day of red and processed red meat [122] alone, i.e., without including additional poultry or fish, eggs or dairy. However, eating less meat and other animal products is increasingly seen as a healthier, more ethical, more sustainable option [123], that is not only “better for you” but also “better for the planet” [124]. Rising awareness of these facts has contributed to shifts in policy focus and public attitudes to meat consumption. For instance, Public Health England’s salt-reduction target reports have featured PBMAs since 2017 [125,126] and the recent European Institute of Innovation & Technology (EIT) Food study “The V-Place-Enabling consumer choice in Vegan or Vegetarian Food Products”—carried out by scientists at the Hohenheim Research Center for Bioeconomy—concluded that approximately 75 million European consumers purchase vegan or vegetarian foods each year and that this trend is rising [127]. Moreover, public health messages around COVID-19 have highlighted the interconnected nature of disease epidemics, food systems, and nutrition [128], emphasising the role of a healthy, well-balanced diet as a pragmatic risk management approach for the support of healthy immunity [129,130,131]. Further, public health messages during the COVID-19 pandemic have highlighted the interconnected nature of disease epidemics, food systems, and nutrition [132,133]. Thus, a growing number of people have increased consumption of plant-based foods and reduced consumption of animal produce. As a result, these are the two most frequently reported changes in eating habits by populations from around the world during the pandemic [134,135,136,137,138,139,140].

One of the outcomes of this systemic change is the growth of flexitarianism as a popular social identity and lifestyle choice. Health- and environment-conscious flexitarians embrace a “quasi vegetarian” dietary pattern that allows for occasional consumption of meat, fish, seafood, poultry and other animal products [141,142]. Beyond the indisputable focus on improving personal health and wellbeing, flexitarian motivations to eat less meat include price, a desire to increase dietary variety and the reduction of social unease when eating with peers who may disapprove of meat eating [143,144]. Other more altruistic motives include concerns about the future of the environment and animal welfare [145,146]. Furthermore, all these reasons for the reduction of meat consumption—or at least the intention thereof—have been embedded as societal norms with powerful effects on our food choice and intake. Eating less meat is increasingly seen as a healthier, more ethical, more sustainable option [147], so people adjust their eating behaviours to manage their public image and “to create a certain impression on others” [148]. Interestingly, recent research suggests that flexitarians are more likely to include plant-based meat alternatives (PBMAs) in their diets [149,150]. Research also shows that flexitarians, in addition to vegetarians and vegans, are more attracted to plant-based meat substitutes that imitate processed meat products, such as burgers, meatballs or sausages, than to those that imitate unprocessed meats, e.g., plant-based steaks [151]. This may be explained by the fact that people are attracted to foods for a range of different reasons, including the pleasure they experience as a result of their taste [152,153,154,155,156]. Realistic-looking and -tasting plant-based alternatives to these meat-containing products provide consumers with a similar experience that is more aligned with their values. Additionally, consumers of PBMAs are more likely to eat other convenience plant-based meals and snacks compared to meat eaters [20]. All of these factors have contributed to an increasing demand for plant-based meat substitutes around the world, a market that continues to flourish at a rate that enabled its growth to a total of USD 3.6 bn in 2020, with a predicted increase in value to USD 4.2 bn by the end of 2021 [157].

Provided they are planned appropriately and include a diversity of high-quality ingredients, plant-based diets can be nutritionally adequate and confer a range of health benefits [158,159,160]. Unfortunately, a plant-based diet is not always a healthy diet, and trying to critically appraise this statement can prove a difficult task because the majority of studies of plant-based diets group all plant foods together. In fact, it was only recently (2016–2017) that researchers from the Department of Nutrition at the Chan School of Public Health at Harvard University published the first studies that demonstrated the divergent effects of healthy and unhealthy plant-based diets on cardiovascular disease and type 2 diabetes risk in adults [161,162], highlighting that plant-based diets are only as healthy as the quality of the foods consumed as part of them. Nonetheless, even these recent “deep dives” into the science of plant-based nutrition have neglected the fact that PBMA products are being consumed daily by millions of people around the world, and that the avoidance of animal-based foods appears to be paired with the introduction of larger amounts of ultra-processed foods containing high levels of calories, refined starches, and unhealthy types of fat, in addition to low fibre and micronutrient levels [20,27,28]. According to the NOVA food classification by Monteiro et al. [24], ultra-processed foods (UPFs) are made to be hyper-palatable and attractive, and to have a long shelf-life for the consumer’s convenience [24]. UPFs have also been documented for their ability to alter the provision of nutrient substrates to colonic bacteria, due to the fact that refined carbohydrates and sugars are digested in the upper portion of the gastrointestinal tract, thereby promoting negative changes to both the composition and the metabolic activity of the gut microbiota [31,32,33,163,164]. Most relevantly, some plant-based meat alternatives (PBMAs) may have reduced levels of ingredients of high nutritional value, thus fitting the UPF definition [29,30].

We disagree with the suggestion that the mere industrial processing of ingredients of vegetable origin makes the resulting PBMA product ultra-processed by default. To test our hypothesis, we posited that the real-world, commercially available plant-based mince, burgers, sausages, sausage patties and meatballs used for the intervention would be able to elicit positive changes in the gut microbiota of participants randomised to the intervention group, compared to those in the control group. Given the growing body of literature documenting the negative impact of UPFs on the human gut microbiome, and the fact that microbial composition and functional features are associated with a range of health markers, we consider the changes in the gut microbiota of participants as evidence to support our argument, namely, that consuming PBMA products as part of a healthy balanced diet can elicit changes in the gut microbiota consistent with positive health outcomes.

As part of the study conceptualisation, we identified a distinct lack of good quality evidence to inform consumers, nutritionists, food scientists and food manufacturers about the impact of PBMAs on human health, and to help researchers across a number of fields—including nutrition, microbiology, endocrinology, mental health and public health—compare these with the effect of animal meat. Moreover, we realised that randomised controlled trials (RCTs) aiming to compare the impact of PBMA products on the gut microbiota of consumers with the impact of animal meat were largely non-existent. To our knowledge, our study is the first RCT conducted with the purpose of establishing that comparison, thus contributing to fill the identified knowledge gap.

### 4.3. Limitations of the Study

We consider the study sample size as the main limitation of the study, and that a larger sample may have provided more clarity about some of the findings that were just slightly above the accepted confidence interval for statistical significance. We are aware of the advantages of a crossover trial design, but finding participants who meet the inclusion criteria and are committed to the study for the duration of the intervention is a difficult task. Health sciences literature deems crossover designs to be superior in terms of data reliability. However, we have been recruiting for several small trials at our laboratory during the past several years and observed lower levels of participant compliance and higher dropout rates in crossover design studies [165]. This becomes much more problematic than in parallel trials from a statistical perspective [166]. In addition, funding for our trial was very limited. Based on these practicalities, we choose a parallel design as a starting point, with views to using a crossover design that incorporates interim sampling, e.g., each 7 to 10 days, in future iterations of the study. Moreover, the analysis of metabolic potential for the synthesis of SCFAs relied on bioinformatic analyses of 16S rRNA raw data. Again, we see this as a starting point, and would hope to retest our hypotheses with the addition of shotgun metagenomic sequencing data.

Another issue that we discussed during the design stage was whether to ask participants to provide detailed food consumption records. Although we acknowledge the value that this data would have added to the study, our team’s focus was to facilitate an experience that was as close to “real-world” as possible for participants. Based on our own experience of using various food reporting questionnaires, participants tend to forget, misestimate and misreport their food consumption, sometimes intentionally, because they may feel embarrassed by the lack of compliance, for example. On that basis, we pondered the potential effect of asking for a detailed food log for 30 days, and decided to evaluate our findings on the basis of the number of meatless meals consumed per week, in addition to the potential side effects reported. We do not rule out the use of a food reporting tool in a future iteration of the study, but are still highly aware of the well-known caveats we mentioned above.

The timing of the study may have posed another potential limitation given that it ran during January, a month when many people attempt healthier diet and lifestyle changes, with a popular campaign known as Veganuary [167] running throughout this first month of the year. We did advise participants to continue to consume the kinds of foods they normally shopped for which, as per our inclusion criteria, were “typically British” [168,169], i.e., including meat, fish, eggs, and dairy most days of the week, if not daily. We did not, however, capture dietary information from the control group, so there is a possibility that some controls might have decided to improve their diet and lifestyle during this period by cutting down on meat and including more fruit, vegetables and other foods of plant origin during this period. We identified another potential source of bias in the control group. The participant information sheet sent to all participants made them aware of the fact that they would be receiving their test results after the study was completed. It is therefore possible that, knowing they would receive before and after results from their microbiome tests, participants in the control group saw this as an opportunity to engage in a dietary change that they did not share with the researchers in order to measure themselves. We are also aware that January is a month when people tend to participate in other kinds of “health kicks”, such as drinking less alcohol or exercising more, both of which are known to have an impact on the gut microbiota [170,171].

## 5. Conclusions 

Our team aimed to test the hypothesis that the mere industrial processing of ingredients of plant origin does not make a PBMA product ultra-processed by default, arguing that the potential for a PBMA product to promote either eubiotic or dysbiotic changes in the gut microbiota of consumers lies in the nutrient profiles of each of its individual ingredients. We performed 16S rRNA sequencing of stool samples provided by participants who replaced ∼5 meals/week containing animal produce with meals containing plant-based mince, meatballs, sausages, sausage patties and burgers, and compared the results to those from samples provided by size-matched control group. Compositionally-aware bioinformatic analysis of before/after data revealed small but statistically significant changes in the presence of butyrate-producing pathways—chiefly the 4-aminobutyrate/succinate and glutarate pathways in the intervention group—in addition to a consistent increase in the joint abundances of butyrate-producing taxa. We also observed a decrease in the Tenericutes phylum and higher beta-diversity between paired samples in the intervention group only, alongside an increase in the Tenericutes phylum in controls. Despite the limitations of a small sample, a parallel trial design, and the reliance on 16S rRNA data only, we were able to confirm that the PBMA products provided to participants in the intervention group elicited changes in their gut microbiota that are consistent with eubiosis, i.e., “a healthy gut microbiome”, meaning that the occasional replacement of animal meats with PBMA products seen in flexitarian dietary patterns may promote positive changes to the gut microbiome of consumers. This provides us with a starting point from which to continue to test our hypotheses with a crossover trial design and the addition of shotgun metabolomics data.

## Figures and Tables

**Table 1:** Intervention products’ macronutrients and protein source.

Per 100 g	Burger	Sausage	Mince	Sausage Patty	Meatballs	Mean ± SD
Calories (kcal)	230	234	199	233	223	227.95 ± 7.07
Protein (grams)	17.1	14.4	19.1	16.6	15	15.23 ± 1.20
Fibre (grams)	3.7	3.2	4.9	2.5	4.2	3.64 ± 0.82
Fat (grams)	14.8	15.9	10.9	15	11.9	11.69 ± 5.16
(of which saturates)	4.7	5	3.9	4.2	0.8	2.00 ± 2.97
Carbohydrate (grams)	5.3	6.9	7.8	10	11.8	6.70 ± 0.28
(of which sugars)	0.3	0.3	0.1	0	0.9	0.30 ± 0.00
Salt (grams)	1.49	1.27	0.62	1.26	1.36	1.38 ± 0.16
Cholesterol (grams)	0	0	0	0	0	
Protein source	Pea and rice	Pea and rice	Soy, pea and rice	Pea	Pea	

Protein, fibre, fat (including saturates), carbohydrate (including sugars) and salt are all reported in grams per 100 g. 2 Saturated fat is monosaturated fat from coconut oil.

**Table 2:** Phytonutrient content in whole pea, pea flour and pea protein concentrate.

Protein Source	Daidzein (mg/kg)	Secoisolariciresonol (mg/kg)	Ferulic Acid (mg/kg)	Vitamin K1 (μg/100 g)	Vitamin K2(MK4) (μg/100 g)	Vitamin K2(MK7)(μg/100 g)	Genistein (mg/kg)	Lutein (mg/kg)	Zeaxanthin (mg/kg)
Pea Flour	<0.5	<0.5	3.5	11.8	0.35	0.17	0.75	1.73	<0.5
Pea Protein Concentrate (Dry Fractionated)	<0.5	<0.5	2.5	12	1.92	2.29	0.85	12.29	<0.5
Dehulled Peas	<0.5	<0.5	2.9	13.6	0.46	0.16	0.15	7.92	<0.5

Independent analysis carried out by Campden BRI (Chipping Campden, Gloucestershire, UK). Vitamin K, ferulic acid and genistein analysed by LC/MS/MS liquid chromatography with mass spectrometry detection. Lutein and zeaxanthin analysed by liquid chromatography with UV detection.

## Data Availability

Datasets, reports for all statistical analyses and microbiome raw data/raw reads are publicly available for download at http://bit.ly/ETH2021-0025a. (main report) and http://bit.ly/ETH2021-0025b (functional analysis report). The online reports contain a wealth of interactive visualisations, such as heatmaps, PCoA-based taxonomic composition, hierarchical clustering, taxa co-occurrence graphs, and metabolic potential graphs, which are also available to anyone wishing to explore these datasets more closely.

## Supplementary Materials

The following are available online at https://www.mdpi.com/article/10.3390/foods10092040/s1. Supplementary materials include 1. Before and after heatmap. 2. Updated ingredients sheet. 3. Meal consumption data and side effects. 4. Anthropometric data (detailed). 4a. Anthropometric data (summarised). 5. Recipe book. 6. Study flow diagram. 7. Pea protein phytonutrient analysis data. 8–12. Product information sheets. 13. Supplementary data visualisations.

## References

[1] You W., Henneberg M. (2016). Meat consumption providing a surplus energy in modern diet contributes to obesity prevalence: An ecological analysis. BMC Nutr..

[2] Montonen J., Boeing H., Fritsche A., Schleicher E., Joost H.-G., Schulze M.B., Steffen A., Pischon T. (2013). Consumption of red meat and whole-grain bread in relation to biomarkers of obesity, inflammation, glucose metabolism and oxidative stress. Eur. J. Nutr..

[3] InterAct Consortium (2013). Association between dietary meat consumption and incident type 2 diabetes: The EPIC-InterAct study. Diabetologia.

[4] Feskens E.J.M., Sluik D., van Woudenbergh G.J. (2013). Meat Consumption, Diabetes, and Its Complications. Curr. Diabetes Rep..

[5] Micha R., Wallace S.K., Mozaffarian D. (2010). Red and Processed Meat Consumption and Risk of Incident Coronary Heart Disease, Stroke, and Diabetes Mellitus. Circulation.

[6] Bechthold A., Boeing H., Schwedhelm C., Hoffmann G., Knüppel S., Iqbal K., De Henauw S., Michels N., Devleesschauwer B., Schlesinger S. (2019). Food groups and risk of coronary heart disease, stroke and heart failure: A systematic review and dose-response meta-analysis of prospective studies. Crit. Rev. Food Sci. Nutr..

[7] Parkin D.M., Boyd L., Walker L.C. (2011). 16. The fraction of cancer attributable to lifestyle and environmental factors in the UK in 2010. Br. J. Cancer.

[8] Bouvard V., Loomis D., Guyton K.Z., Grosse Y., Ghissassi F.E., Benbrahim-Tallaa L., Guha N., Mattock H., Straif K. (2015). Carcinogenicity of consumption of red and processed meat. Lancet Oncol..

[9] Boeing H., Bechthold A., Bub A., Ellinger S., Haller D., Kroke A., Leschik-Bonnet E., Müller M.J., Oberritter H., Schulze M. (2012). Critical review: Vegetables and fruit in the prevention of chronic diseases. Eur. J. Nutr..

[10] Wang X., Ouyang Y., Liu J., Zhu M., Zhao G., Bao W., Hu F.B. (2014). Fruit and vegetable consumption and mortality from all causes, cardiovascular disease, and cancer: Systematic review and dose-response meta-analysis of prospective cohort studies. BMJ.

[11] Sakkas H., Bozidis P., Touzios C., Kolios D., Athanasiou G., Athanasopoulou E., Gerou I., Gartzonika C. (2020). Nutritional Status and the Influence of the Vegan Diet on the Gut Microbiota and Human Health. Medicina.

[12] Medawar E., Huhn S., Villringer A., Veronica Witte A. (2019). The effects of plant-based diets on the body and the brain: A systematic review. Transl. Psychiatry.

[13] Barber T.M., Kabisch S., Pfeiffer A.F.H., Weickert M.O. (2020). The Health Benefits of Dietary Fibre. Nutrients.

[14] Tomova A., Bukovsky I., Rembert E., Yonas W., Alwarith J., Barnard N.D., Kahleova H. (2019). The Effects of Vegetarian and Vegan Diets on Gut Microbiota. Front. Nutr..

[15] Wilson A.S., Koller K.R., Ramaboli M.C., Nesengani L.T., Ocvirk S., Chen C., Flanagan C.A., Sapp F.R., Merritt Z.T., Bhatti F. (2020). Diet and the Human Gut Microbiome: An International Review. Dig. Dis. Sci..

[16] Boukid F., Rosell C.M., Rosene S., Bover-Cid S., Castellari M. (2021). Non-animal proteins as cutting-edge ingredients to reformulate animal-free foodstuffs: Present status and future perspectives. Crit. Rev. food Sci. Nutr..

[17] Chen C., Chaudhary A., Mathys A. (2019). Dietary Change Scenarios and Implications for Environmental, Nutrition, Human Health and Economic Dimensions of Food Sustainability. Nutrients.

[18] Curtain F., Grafenauer S. (2019). Plant-Based Meat Substitutes in the Flexitarian Age: An Audit of Products on Supermarket Shelves. Nutrients.

[19] Michel F., Hartmann C., Siegrist M. (2021). Consumers’ associations, perceptions and acceptance of meat and plant-based meat alternatives. Food Qual. Prefer..

[20] Gallagher C.T., Hanley P., Lane K.E. (2021). Pattern analysis of vegan eating reveals healthy and unhealthy patterns within the vegan diet. Public Health Nutr..

[21] Saswattecha K., Kroeze C., Jawjit W., Hein L. (2015). Assessing the environmental impact of palm oil produced in Thailand. J. Clean. Prod..

[22] Wahyono Y., Hadiyanto H., Budihardjo M.A., Adiansyah J.S. (2020). Assessing the Environmental Performance of Palm Oil Biodiesel Production in Indonesia: A Life Cycle Assessment Approach. Energies.

[23] Boccia F., Punzo G. (2021). A choice experiment on consumer perceptions of three generations of genetically modified foods. Appetite.

[24] Monteiro C.A., Cannon G., Moubarac J.C., Levy R.B., Louzada M.L.C., Jaime P.C. (2018). The UN Decade of Nutrition, the NOVA food classification and the trouble with ultra-processing. Public Health Nutr..

[25] Clune S., Crossin E., Verghese K. (2017). Systematic review of greenhouse gas emissions for different fresh food categories. J. Clean. Prod..

[26] Soret S., Mejia A., Batech M., Jaceldo-Siegl K., Harwatt H., Sabaté J. (2014). Climate change mitigation and health effects of varied dietary patterns in real-life settings throughout North America. Am. J. Clin. Nutr..

[27] Alcorta A., Porta A., Tárrega A., Alvarez M.D., Vaquero M.P. (2021). Foods for Plant-Based Diets: Challenges and Innovations. Foods.

[28] Gehring J., Touvier M., Baudry J., Julia C., Buscail C., Srour B., Hercberg S., Péneau S., Kesse-Guyot E., Allès B. (2021). Consumption of Ultra-Processed Foods by Pesco-Vegetarians, Vegetarians, and Vegans: Associations with Duration and Age at Diet Initiation. J. Nutr..

[29] Bohrer B.M. (2019). An investigation of the formulation and nutritional composition of modern meat analogue products. Food Sci. Hum. Wellness.

[30] Thavamani A., Sferra T.J., Sankararaman S. (2020). Meet the Meat Alternatives: The Value of Alternative Protein Sources. Curr. Nutr. Rep..

[31] Dahl W.J., Rivero Mendoza D., Lambert J.M. (2020). Diet, nutrients and the microbiome. Prog. Mol. Biol. Transl. Sci..

[32] Martínez Leo E.E., Segura Campos M.R. (2020). Effect of ultra-processed diet on gut microbiota and thus its role in neurodegenerative diseases. Nutrition.

[33] Zinöcker M.K., Lindseth I.A. (2018). The Western Diet-Microbiome-Host Interaction and Its Role in Metabolic Disease. Nutrients.

[34] Kamm M.A. (2020). Processed food affects the gut microbiota: The revolution has started. J. Gastroenterol. Hepatol..

[35] Create a Blocked Randomisation List. https://www.sealedenvelope.com/simple-randomiser/v1/lists.

[36] Farm M. Meatless Farm-Make It Meatless!. https://meatlessfarm.com/.

[37] Corp I. (2017). IBM SPSS Statistics for Windows, Version 25.0.

[38] Biomed A. The Atlas Biomed Microbiome Test. https://atlasbiomed.com/uk/microbiome.

[39] Panek M., Čipčić Paljetak H., Barešić A., Perić M., Matijašić M., Lojkić I., Vranešić Bender D., Krznarić Ž., Verbanac D. (2018). Methodology challenges in studying human gut microbiota-effects of collection, storage, DNA extraction and next generation sequencing technologies. Sci. Rep..

[40] Park C., Yun K.E., Chu J.M., Lee J.Y., Hong C.P., Nam Y.D., Jeong J., Han K., Ahn Y.J. (2020). Performance comparison of fecal preservative and stock solutions for gut microbiome storage at room temperature. J. Microbiol..

[41] Biomed A. About Atlas. https://atlasbiomed.com/uk/about.

[42] ISO (2016). ISO 13485:2016(en), Medical Devices—Quality Management Systems. Requirements for Regulatory Purposes.

[43] Chen C.C., Wu W.K., Chang C.M., Panyod S., Lu T.P., Liou J.M., Fang Y.J., Chuang E.Y., Wu M.S. (2020). Comparison of DNA stabilizers and storage conditions on preserving fecal microbiota profiles. J. Formos. Med. Assoc..

[44] Caporaso J.G., Lauber C.L., Walters W.A., Berg-Lyons D., Huntley J., Fierer N., Owens S.M., Betley J., Fraser L., Bauer M. (2012). Ultra-high-throughput microbial community analysis on the Illumina HiSeq and MiSeq platforms. ISME J..

[45] Ravi R.K., Walton K., Khosroheidari M. (2018). MiSeq: A Next Generation Sequencing Platform for Genomic Analysis. Methods Mol. Biol..

[46] Amir A., McDonald D., Navas-Molina J.A., Kopylova E., Morton J.T., Zech Xu Z., Kightley E.P., Thompson L.R., Hyde E.R., Gonzalez A. (2017). Deblur Rapidly Resolves Single-Nucleotide Community Sequence Patterns. mSystems.

[47] Bolyen E., Rideout J.R., Dillon M.R., Bokulich N.A., Abnet C.C., Al-Ghalith G.A., Alexander H., Alm E.J., Arumugam M., Asnicar F. (2019). Reproducible, interactive, scalable and extensible microbiome data science using QIIME 2. Nat. Biotechnol..

[48] DeSantis T.Z., Hugenholtz P., Larsen N., Rojas M., Brodie E.L., Keller K., Huber T., Dalevi D., Hu P., Andersen G.L. (2006). Greengenes, a Chimera-Checked 16S rRNA Gene Database and Workbench Compatible with ARB. Appl. Environ. Microbiol..

[49] Wagner B.D., Grunwald G.K., Zerbe G.O., Mikulich-Gilbertson S.K., Robertson C.E., Zemanick E.T., Harris J.K. (2018). On the Use of Diversity Measures in Longitudinal Sequencing Studies of Microbial Communities. Front. Microbiol..

[50] Willis A.D. (2019). Rarefaction, Alpha Diversity, and Statistics. Front. Microbiol..

[51] Maziarz M., Pfeiffer R.M., Wan Y., Gail M.H. (2018). Using standard microbiome reference groups to simplify beta-diversity analyses and facilitate independent validation. Bioinformatics.

[52] Modin O., Liébana R., Saheb-Alam S., Wilén B.-M., Suarez C., Hermansson M., Persson F. (2020). Hill-based dissimilarity indices and null models for analysis of microbial community assembly. Microbiome.

[53] Palarea-Albaladejo J., Martín-Fernández J.A. (2015). zCompositions—R package for multivariate imputation of left-censored data under a compositional approach. Chemom. Intell. Lab. Syst..

[54] Quinn T.P., Erb I. (2020). Interpretable Log Contrasts for the Classification of Health Biomarkers: A New Approach to Balance Selection. mSystems.

[55] Haynes W., Dubitzky W., Wolkenhauer O., Cho K.-H., Yokota H. (2013). Benjamini–Hochberg Method. Encyclopedia of Systems Biology.

[56] Takahashi K., Nishida A., Fujimoto T., Fujii M., Shioya M., Imaeda H., Inatomi O., Bamba S., Sugimoto M., Andoh A. (2016). Reduced Abundance of Butyrate-Producing Bacteria Species in the Fecal Microbial Community in Crohn’s Disease. Digestion.

[57] Gloor G. (2015). ALDEx2: ANOVA-Like Differential Expression tool for compositional data. ALDEX Man. Modul..

[58] Caporaso J.G., Kuczynski J., Stombaugh J., Bittinger K., Bushman F.D., Costello E.K. (2010). QIIME allows analysis of high-throughput community sequencing data. Nat. Methods.

[59] Langille M.G.I., Zaneveld J., Caporaso J.G., McDonald D., Knights D., Reyes J.A., Clemente J.C., Burkepile D.E., Vega Thurber R.L., Knight R. (2013). Predictive functional profiling of microbial communities using 16S rRNA marker gene sequences. Nat. Biotechnol..

[60] Efimova D., Tyakht A., Popenko A., Vasilyev A., Altukhov I., Dovidchenko N., Odintsova V., Klimenko N., Loshkarev R., Pashkova M. (2018). Knomics-Biota—A system for exploratory analysis of human gut microbiota data. BioData Min..

[61] Gonlachanvit S., Coleski R., Owyang C., Hasler W. (2004). Inhibitory actions of a high fibre diet on intestinal gas transit in healthy volunteers. Gut.

[62] Klimenko N.S., Tyakht A.V., Popenko A.S., Vasiliev A.S., Altukhov I.A., Ischenko D.S., Shashkova T.I., Efimova D.A., Nikogosov D.A., Osipenko D.A. (2018). Microbiome Responses to an Uncontrolled Short-Term Diet Intervention in the Frame of the Citizen Science Project. Nutrients.

[63] StataCorp Stata|New in Stata. https://www.stata.com/new-in-stata/.

[64] Lin H., Peddada S.D. (2020). Analysis of microbial compositions: A review of normalization and differential abundance analysis. NPJ Biofilms Microbiomes.

[65] Weiss S., Xu Z.Z., Peddada S., Amir A., Bittinger K., Gonzalez A., Lozupone C., Zaneveld J.R., Vázquez-Baeza Y., Birmingham A. (2017). Normalization and microbial differential abundance strategies depend upon data characteristics. Microbiome.

[66] Kurtz Z.D., Müller C.L., Miraldi E.R., Littman D.R., Blaser M.J., Bonneau R.A. (2015). Sparse and Compositionally Robust Inference of Microbial Ecological Networks. PLoS Comput. Biol..

[67] Egozcue J.J., Pawlowsky-Glahn V. (2005). Groups of Parts and Their Balances in Compositional Data Analysis. Math. Geol..

[68] Duvallet C., Gibbons S.M., Gurry T., Irizarry R.A., Alm E.J. (2017). Meta-analysis of gut microbiome studies identifies disease-specific and shared responses. Nat. Commun..

[69] Rackerby B., Kim H.J., Dallas D.C., Park S.H. (2020). Understanding the effects of dietary components on the gut microbiome and human health. Food Sci. Biotechnol..

[70] Nagpal R., Shively C.A., Register T.C., Craft S., Yadav H. (2019). Gut microbiome-Mediterranean diet interactions in improving host health. F1000Research.

[71] Wang D.D., Nguyen L.H., Li Y., Yan Y., Ma W., Rinott E., Ivey K.L., Shai I., Willett W.C., Hu F.B. (2021). The gut microbiome modulates the protective association between a Mediterranean diet and cardiometabolic disease risk. Nat. Med..

[72] Louis P., Flint H.J. (2009). Diversity, metabolism and microbial ecology of butyrate-producing bacteria from the human large intestine. FEMS Microbiol. Lett..

[73] Patman G. (2015). Lactobacillus acidophilus opens the door to butyrate. Nat. Rev. Gastroenterol. Hepatol..

[74] Rivière A., Selak M., Lantin D., Leroy F., De Vuyst L. (2016). Bifidobacteria and Butyrate-Producing Colon Bacteria: Importance and Strategies for Their Stimulation in the Human Gut. Front. Microbiol..

[75] Markowiak-Kopeć P., Śliżewska K. (2020). The Effect of Probiotics on the Production of Short-Chain Fatty Acids by Human Intestinal Microbiome. Nutrients.

[76] Rothschild D., Leviatan S., Hanemann A., Cohen Y., Weissbrod O., Segal E. (2020). An atlas of robust microbiome associations with phenotypic traits based on large-scale cohorts from two continents. bioRxiv.

[77] Falony G., Joossens M., Vieira-Silva S., Wang J., Darzi Y., Faust K., Kurilshikov A., Bonder M.J., Valles-Colomer M., Vandeputte D. (2016). Population-level analysis of gut microbiome variation. Science.

[78] Coppola S., Avagliano C., Calignano A., Berni Canani R. (2021). The Protective Role of Butyrate against Obesity and Obesity-Related Diseases. Molecules.

[79] McNabney S.M., Henagan T.M. (2017). Short Chain Fatty Acids in the Colon and Peripheral Tissues: A Focus on Butyrate, Colon Cancer, Obesity and Insulin Resistance. Nutrients.

[80] Sivaprakasam S., Bhutia Y.D., Yang S., Ganapathy V. (2017). Short-Chain Fatty Acid Transporters: Role in Colonic Homeostasis. Compr. Physiol..

[81] Wong J.M., de Souza R., Kendall C.W., Emam A., Jenkins D.J. (2006). Colonic health: Fermentation and short chain fatty acids. J. Clin. Gastroenterol..

[82] Ashaolu T.J., Ashaolu J.O., Adeyeye S.A.O. (2021). Fermentation of prebiotics by human colonic microbiota in vitro and short-chain fatty acids production: A critical review. J. Appl. Microbiol..

[83] Velázquez O.C., Lederer H.M., Rombeau J.L. (1996). Butyrate and the colonocyte. Dig. Dis. Sci..

[84] Zhang L., Liu C., Jiang Q., Yin Y. (2021). Butyrate in Energy Metabolism: There Is Still More to Learn. Trends Endocrinol. Metab..

[85] Qin J., Li Y., Cai Z., Li S., Zhu J., Zhang F., Liang S., Zhang W., Guan Y., Shen D. (2012). A metagenome-wide association study of gut microbiota in type 2 diabetes. Nature.

[86] Le Chatelier E., Nielsen T., Qin J., Prifti E., Hildebrand F., Falony G., Almeida M., Arumugam M., Batto J.M., Kennedy S. (2013). Richness of human gut microbiome correlates with metabolic markers. Nature.

[87] Karlsson F.H., Fåk F., Nookaew I., Tremaroli V., Fagerberg B., Petranovic D., Bäckhed F., Nielsen J. (2012). Symptomatic atherosclerosis is associated with an altered gut metagenome. Nat. Commun..

[88] Vital M., Karch A., Pieper D.H. (2017). Colonic Butyrate-Producing Communities in Humans: An Overview Using Omics Data. mSystems.

[89] Rivera-Chávez F., Zhang L.F., Faber F., Lopez C.A., Byndloss M.X., Olsan E.E., Xu G., Velazquez E.M., Lebrilla C.B., Winter S.E. (2016). Depletion of Butyrate-Producing Clostridia from the Gut Microbiota Drives an Aerobic Luminal Expansion of Salmonella. Cell Host Microbe.

[90] Pavel F.M., Vesa C.M., Gheorghe G., Diaconu C.C., Stoicescu M., Munteanu M.A., Babes E.E., Tit D.M., Toma M.M., Bungau S. (2021). Highlighting the Relevance of Gut Microbiota Manipulation in Inflammatory Bowel Disease. Diagnostics.

[91] Prosberg M., Bendtsen F., Vind I., Petersen A.M., Gluud L.L. (2016). The association between the gut microbiota and the inflammatory bowel disease activity: A systematic review and meta-analysis. Scand. J. Gastroenterol..

[92] Oliphant K., Allen-Vercoe E. (2019). Macronutrient metabolism by the human gut microbiome: Major fermentation by-products and their impact on host health. Microbiome.

[93] Louis P., Flint H.J. (2017). Formation of propionate and butyrate by the human colonic microbiota. Environ. Microbiol..

[94] Louis P., Young P., Holtrop G., Flint H.J. (2010). Diversity of human colonic butyrate-producing bacteria revealed by analysis of the butyryl-CoA:acetate CoA-transferase gene. Environ. Microbiol..

[95] Vital M., Howe A.C., Tiedje J.M. (2014). Revealing the bacterial butyrate synthesis pathways by analyzing (meta) genomic data. MBio.

[96] Haro C., Montes-Borrego M., Rangel-Zúñiga O.A., Alcalá-Díaz J.F., Gómez-Delgado F., Pérez-Martínez P., Delgado-Lista J., Quintana-Navarro G.M., Tinahones F.J., Landa B.B. (2016). Two Healthy Diets Modulate Gut Microbial Community Improving Insulin Sensitivity in a Human Obese Population. J. Clin. Endocrinol. Metab..

[97] Rosés C., Cuevas-Sierra A., Quintana S., Riezu-Boj J.I., Martínez J.A., Milagro F.I., Barceló A. (2021). Gut Microbiota Bacterial Species Associated with Mediterranean Diet-Related Food Groups in a Northern Spanish Population. Nutrients.

[98] Meslier V., Laiola M., Roager H.M., De Filippis F., Roume H., Quinquis B., Giacco R., Mennella I., Ferracane R., Pons N. (2020). Mediterranean diet intervention in overweight and obese subjects lowers plasma cholesterol and causes changes in the gut microbiome and metabolome independently of energy intake. Gut.

[99] Kahleova H., Rembert E., Alwarith J., Yonas W.N., Tura A., Holubkov R., Agnello M., Chutkan R., Barnard N.D. (2020). Effects of a Low-Fat Vegan Diet on Gut Microbiota in Overweight Individuals and Relationships with Body Weight, Body Composition, and Insulin Sensitivity. A Randomized Clinical Trial. Nutrients.

[100] De Filippis F., Pasolli E., Ercolini D. (2020). Newly Explored Faecalibacterium Diversity Is Connected to Age, Lifestyle, Geography, and Disease. Curr. Biol..

[101] Shevlyakov A., Nikogosov D., Stewart L.-A., Toribio-Mateas M. (2021). Reference values for intake of 6 types of soluble and insoluble fibre in healthy UK inhabitants based on the UK Biobank data. Public Health Nutr..

[102] Molan A.L., Liu Z., Plimmer G. (2014). Evaluation of the effect of blackcurrant products on gut microbiota and on markers of risk for colon cancer in humans. Phytother. Res..

[103] Dingeo G., Brito A., Samouda H., Iddir M., La Frano M.R., Bohn T. (2020). Phytochemicals as modifiers of gut microbial communities. Food Funct..

[104] Song Y., Wu M.-S., Tao G., Lu M.-W., Lin J., Huang J.-Q. (2020). Feruloylated oligosaccharides and ferulic acid alter gut microbiome to alleviate diabetic syndrome. Food Res. Int..

[105] Corona G., Kreimes A., Barone M., Turroni S., Brigidi P., Keleszade E., Costabile A. (2020). Impact of lignans in oilseed mix on gut microbiome composition and enterolignan production in younger healthy and premenopausal women: An in vitro pilot study. Microb. Cell Factories.

[106] David L.A., Maurice C.F., Carmody R.N., Gootenberg D.B., Button J.E., Wolfe B.E. (2014). Diet rapidly and reproducibly alters the human gut microbiome. Nature.

[107] Bonder M.J., Tigchelaar E.F., Cai X., Trynka G., Cenit M.C., Hrdlickova B., Zhong H., Vatanen T., Gevers D., Wijmenga C. (2016). The influence of a short-term gluten-free diet on the human gut microbiome. Genome Med..

[108] Patrone V., Minuti A., Lizier M., Miragoli F., Lucchini F., Trevisi E., Rossi F., Callegari M.L. (2018). Differential effects of coconut versus soy oil on gut microbiota composition and predicted metabolic function in adult mice. BMC Genomics.

[109] Chen P.Y., Li S., Koh Y.C., Wu J.C., Yang M.J., Ho C.T., Pan M.H. (2019). Oolong Tea Extract and Citrus Peel Polymethoxyflavones Reduce Transformation of l-Carnitine to Trimethylamine-N-Oxide and Decrease Vascular Inflammation in l-Carnitine Feeding Mice. J. Agric. Food Chem..

[110] Wu W.-K., Panyod S., Liu P.-Y., Chen C.-C., Kao H.-L., Chuang H.-L., Chen Y.-H., Zou H.-B., Kuo H.-C., Kuo C.-H. (2020). Characterization of TMAO productivity from carnitine challenge facilitates personalized nutrition and microbiome signatures discovery. Microbiome.

[111] Liu Z.Y., Tan X.Y., Li Q.J., Liao G.C., Fang A.P., Zhang D.M., Chen P.Y., Wang X.Y., Luo Y., Long J.A. (2018). Trimethylamine N-oxide, a gut microbiota-dependent metabolite of choline, is positively associated with the risk of primary liver cancer: A case-control study. Nutr. Metab..

[112] Johnson K.V.A., Burnet P.W.J. (2016). Microbiome: Should we diversify from diversity?. Gut Microbes.

[113] Ackoff R.L., Gharajedaghi J. (1996). Reflections on systems and their models. Syst. Res..

[114] Sturmberg J.P. (2021). Health and Disease Are Dynamic Complex-Adaptive States Implications for Practice and Research. Front. Psychiatry.

[115] Johnson A.J., Vangay P., Al-Ghalith G.A., Hillmann B.M., Ward T.L., Shields-Cutler R.R., Kim A.D., Shmagel A.K., Syed A.N., Walter J. (2019). Daily Sampling Reveals Personalized Diet-Microbiome Associations in Humans. Cell Host Microbe.

[116] Ventriglio A., Sancassiani F., Contu M.P., Latorre M., Di Slavatore M., Fornaro M., Bhugra D. (2020). Mediterranean Diet and its Benefits on Health and Mental Health: A Literature Review. Clin. Pract. Epidemiol. Ment. Health.

[117] Sofi F., Abbate R., Gensini G.F., Casini A. (2010). Accruing evidence on benefits of adherence to the Mediterranean diet on health: An updated systematic review and meta-analysis. Am. J. Clin. Nutr..

[118] Sánchez-Sánchez M.L., García-Vigara A., Hidalgo-Mora J.J., García-Pérez M., Tarín J., Cano A. (2020). Mediterranean diet and health: A systematic review of epidemiological studies and intervention trials. Maturitas.

[119] Mancini J.G., Filion K.B., Atallah R., Eisenberg M.J. (2016). Systematic Review of the Mediterranean Diet for Long-Term Weight Loss. Am. J. Med..

[120] Guasch-Ferré M., Salas-Salvadó J., Ros E., Estruch R., Corella D., Fitó M., Martínez-González M.A. (2017). The PREDIMED trial, Mediterranean diet and health outcomes: How strong is the evidence?. Nutr. Metab. Cardiovasc. Dis..

[121] Beardsworth A., Bryman A. (2004). Meat consumption and meat avoidance among young people. Br. Food J..

[122] Hobbs-Grimmer D.A., Givens D.I., Lovegrove J.A. (2021). Associations between red meat, processed red meat and total red and processed red meat consumption, nutritional adequacy and markers of health and cardio-metabolic diseases in British adults: A cross-sectional analysis using data from UK National Diet and Nutrition Survey. Eur. J. Nutr..

[123] Stoll-Kleemann S., Schmidt U.J. (2017). Reducing meat consumption in developed and transition countries to counter climate change and biodiversity loss: A review of influence factors. Reg. Environ. Chang..

[124] Boukid F. (2021). Plant-based meat analogues: From niche to mainstream. Eur. Food Res. Technol..

[125] Public Health England (2017). Salt Targets 2017: Second Progress Report.

[126] Public Health England (2020). Salt Reduction Targets for 2024.

[127] Gebhardt B. (2021). Plant-Based for the Future. Insights on European Consumer and Expert Opinions.

[128] Gillespie S. (2020). Epidemics and food systems: What gets framed, gets done. Food Secur..

[129] Gasmi A., Noor S., Tippairote T., Dadar M., Menzel A., Bjørklund G. (2020). Individual risk management strategy and potential therapeutic options for the COVID-19 pandemic. Clin. Immunol..

[130] Bousquet J., Anto J.M., Iaccarino G., Czarlewski W., Haahtela T., Anto A., Akdis C.A., Blain H., Canonica G.W., Cardona V. (2020). Is diet partly responsible for differences in COVID-19 death rates between and within countries?. Clin. Transl. Allergy.

[131] Maggini S., Pierre A., Calder P.C. (2018). Immune Function and Micronutrient Requirements Change over the Life Course. Nutrients.

[132] Sanderson Bellamy A., Furness E., Nicol P., Pitt H., Taherzadeh A. (2021). Shaping more resilient and just food systems: Lessons from the COVID-19 Pandemic. Ambio.

[133] Keenan J.M. (2020). COVID, resilience, and the built environment. Environ. Syst. Decis..

[134] Tavakol Z., Ghannadi S., Tabesh M.R., Halabchi F., Noormohammadpour P., Akbarpour S., Alizadeh Z., Nezhad M.H., Reyhan S.K. (2021). Relationship between physical activity, healthy lifestyle and COVID-19 disease severity; a cross-sectional study. J. Public Health.

[135] Wang J., Yeoh E.K., Yung T.K.C., Wong M.C.S., Dong D., Chen X., Chan M.K.Y., Wong E.L.Y., Wu Y., Guo Z. (2021). Change in eating habits and physical activities before and during the COVID-19 pandemic in Hong Kong: A cross-sectional study via random telephone survey. J. Int. Soc. Sports Nutr..

[136] Di Renzo L., Gualtieri P., Pivari F., Soldati L., Attinà A., Cinelli G., Leggeri C., Caparello G., Barrea L., Scerbo F. (2020). Eating habits and lifestyle changes during COVID-19 lockdown: An Italian survey. J. Transl. Med..

[137] Abouzid M., El-Sherif D.M., Eltewacy N.K., Dahman N.B.H., Okasha S.A., Ghozy S., Islam S.M.S., Elburki A.R.F., Ali A.A.M., Hasan M.A. (2021). Influence of COVID-19 on lifestyle behaviors in the Middle East and North Africa Region: A survey of 5896 individuals. J. Transl. Med..

[138] Kolokotroni O., Mosquera M.C., Quattrocchi A., Heraclides A., Demetriou C., Philippou E. (2021). Lifestyle habits of adults during the COVID-19 pandemic lockdown in Cyprus: Evidence from a cross-sectional study. BMC Public Health.

[139] Abdulah D.M., Hassan A.B. (2020). Relation of Dietary Factors with Infection and Mortality Rates of COVID-19 across the World. J. Nutr. Health Aging.

[140] Ogueji I.A., Okoloba M.M., Demoko Ceccaldi B.M. (2021). Coping strategies of individuals in the United Kingdom during the COVID-19 pandemic. Curr. Psychol..

[141] Verain M., Dagevos H., Antonides G., Reisch L., Thogersen J. (2015). Flexitarianism: A range of sustainable food styles. Handbook of Research on Sustainable Consumption.

[142] Derbyshire E.J. (2016). Flexitarian Diets and Health: A Review of the Evidence-Based Literature. Front. Nutr..

[143] Kemper J.A., White S.K. (2021). Young adults’ experiences with flexitarianism: The 4Cs. Appetite.

[144] Plante C.N., Rosenfeld D.L., Plante M., Reysen S. (2019). The role of social identity motivation in dietary attitudes and behaviors among vegetarians. Appetite.

[145] Dakin B.C., Ching A.E., Teperman E., Klebl C., Moshel M., Bastian B. (2021). Prescribing vegetarian or flexitarian diets leads to sustained reduction in meat intake. Appetite.

[146] Mintel (2017). Meat-Free Foods-UK-May 2017-Market Research Report.

[147] Bianchi F., Dorsel C., Garnett E., Aveyard P., Jebb S.A. (2018). Interventions targeting conscious determinants of human behaviour to reduce the demand for meat: A systematic review with qualitative comparative analysis. Int. J. Behav. Nutr. Phys. Act..

[148] Higgs S. (2015). Social norms and their influence on eating behaviours. Appetite.

[149] Chen C., Chaudhary A., Mathys A. (2020). Nutritional and environmental losses embedded in global food waste. Resour. Conserv. Recycl..

[150] Dagevos H. (2021). Finding flexitarians: Current studies on meat eaters and meat reducers. Trends Food Sci. Technol..

[151] Michel F., Sanchez-Siles L.M., Siegrist M. (2021). Predicting how consumers perceive the naturalness of snacks: The usefulness of a simple index. Food Qual. Prefer..

[152] Phan U.T., Chambers E.T. (2016). Motivations for choosing various food groups based on individual foods. Appetite.

[153] Hess J.M., Jonnalagadda S.S., Slavin J.L. (2016). What Is a Snack, Why Do We Snack, and How Can We Choose Better Snacks? A Review of the Definitions of Snacking, Motivations to Snack, Contributions to Dietary Intake, and Recommendations for Improvement. Adv. Nutr..

[154] Yates L., Warde A. (2015). The evolving content of meals in Great Britain. Results of a survey in 2012 in comparison with the 1950s. Appetite.

[155] Chandler P.D., Balasubramanian R., Paynter N., Giulianini F., Fung T., Tinker L.F., Snetselaar L., Liu S., Eaton C., Tobias D.K. (2020). Metabolic signatures associated with Western and Prudent dietary patterns in women. Am. J. Clin. Nutr..

[156] Clatici V.G., Voicu C., Voaides C., Roseanu A., Icriverzi M., Jurcoane S. (2018). Diseases of Civilization—Cancer, Diabetes, Obesity and Acne—the Implication of Milk, IGF-1 and mTORC1. Maedica.

[157] (2021). Global Plant Based Meat Market—Analysis by Source, by Product, by Region, by Country (2020 Edition): Market Insights, COVID-19 Impact, Competition and Forecast (2020–2025).

[158] Song M., Fung T.T., Hu F.B., Willett W.C., Longo V.D., Chan A.T., Giovannucci E.L. (2016). Association of Animal and Plant Protein Intake With All-Cause and Cause-Specific Mortality. JAMA Intern. Med..

[159] Mariotti F. (2019). Animal and Plant Protein Sources and Cardiometabolic Health. Adv. Nutr..

[160] Hemler E.C., Hu F.B. (2019). Plant-Based Diets for Cardiovascular Disease Prevention: All Plant Foods Are Not Created Equal. Curr. Atheroscler. Rep..

[161] Satija A., Bhupathiraju S.N., Rimm E.B., Spiegelman D., Chiuve S.E., Borgi L., Willett W.C., Manson J.E., Sun Q., Hu F.B. (2016). Plant-Based Dietary Patterns and Incidence of Type 2 Diabetes in US Men and Women: Results from Three Prospective Cohort Studies. PLoS Med..

[162] Satija A., Bhupathiraju S.N., Spiegelman D., Chiuve S.E., Manson J.E., Willett W., Rexrode K.M., Rimm E.B., Hu F.B. (2017). Healthful and Unhealthful Plant-Based Diets and the Risk of Coronary Heart Disease in U.S. Adults. J. Am. Coll. Cardiol..

[163] Lane M., Howland G., West M., Hockey M., Marx W., Loughman A., O’Hely M., Jacka F., Rocks T. (2020). The effect of ultra-processed very low-energy diets on gut microbiota and metabolic outcomes in individuals with obesity: A systematic literature review. Obes. Res. Clin. Pract..

[164] Sandall A.M., Cox S.R., Lindsay J.O., Gewirtz A.T., Chassaing B., Rossi M., Whelan K. (2020). Emulsifiers Impact Colonic Length in Mice and Emulsifier Restriction is Feasible in People with Crohn’s Disease. Nutrients.

[165] Miranda J., Portocarrero A., Freire A., Abuin C., Saez A. (2020). Advantages, Disadvantages, and Future Trends on the Use of Design of Experiments in Cross-Over Trials in Nutritional Clinical Investigation.

[166] Mills E.J., Chan A.-W., Wu P., Vail A., Guyatt G.H., Altman D.G. (2009). Design, analysis, and presentation of crossover trials. Trials.

[167] Veganuary. Veganuary—The International Movement Inspiring People to Try Vegan!.

[168] Evans C.E.L. (2020). Dietary fibre and cardiovascular health: A review of current evidence and policy. Proc. Nutr. Soc..

[169] Livesey G., Taylor R., Livesey H.F., Buyken A.E., Jenkins D.J.A., Augustin L.S.A., Sievenpiper J.L., Barclay A.W., Liu S., Wolever T.M.S. (2019). Dietary Glycemic Index and Load and the Risk of Type 2 Diabetes: A Systematic Review and Updated Meta-Analyses of Prospective Cohort Studies. Nutrients.

[170] Litwinowicz K., Choroszy M., Waszczuk E. (2020). Changes in the composition of the human intestinal microbiome in alcohol use disorder: A systematic review. Am. J. Drug Alcohol. Abus..

[171] Ortiz-Alvarez L., Xu H., Martinez-Tellez B. (2020). Influence of Exercise on the Human Gut Microbiota of Healthy Adults: A Systematic Review. Clin. Transl. Gastroenterol..

