# Supplementary material for: Impact of Plant-Based Meat Alternatives on the Gut Microbiota of Consumers: A Real-World Study
Source: Foods. 2021 Aug 30;10(9):2040. doi: 10.3390/foods10092040 (PMC8465665; doi:10.3390/foods10092040)
Supplement: Supplementary file 1 [file foods-10-02040-s001.zip › 13. FOODS LBSU ETH2021-0025. Supplementary data 1.pdf]

Appendix A

The following diagrammatic representations of the study data are provided for the benefit of readers and fellow researchers. A whole array of visualisations are available from the Knomics Biota bioinformatics plaform, many of which are interactive. Readers are encouraged to find these on http://bit.ly/ETH2021-0025a. (main report) and http://bit.ly/ETH2021-0025b (functional analysis report).

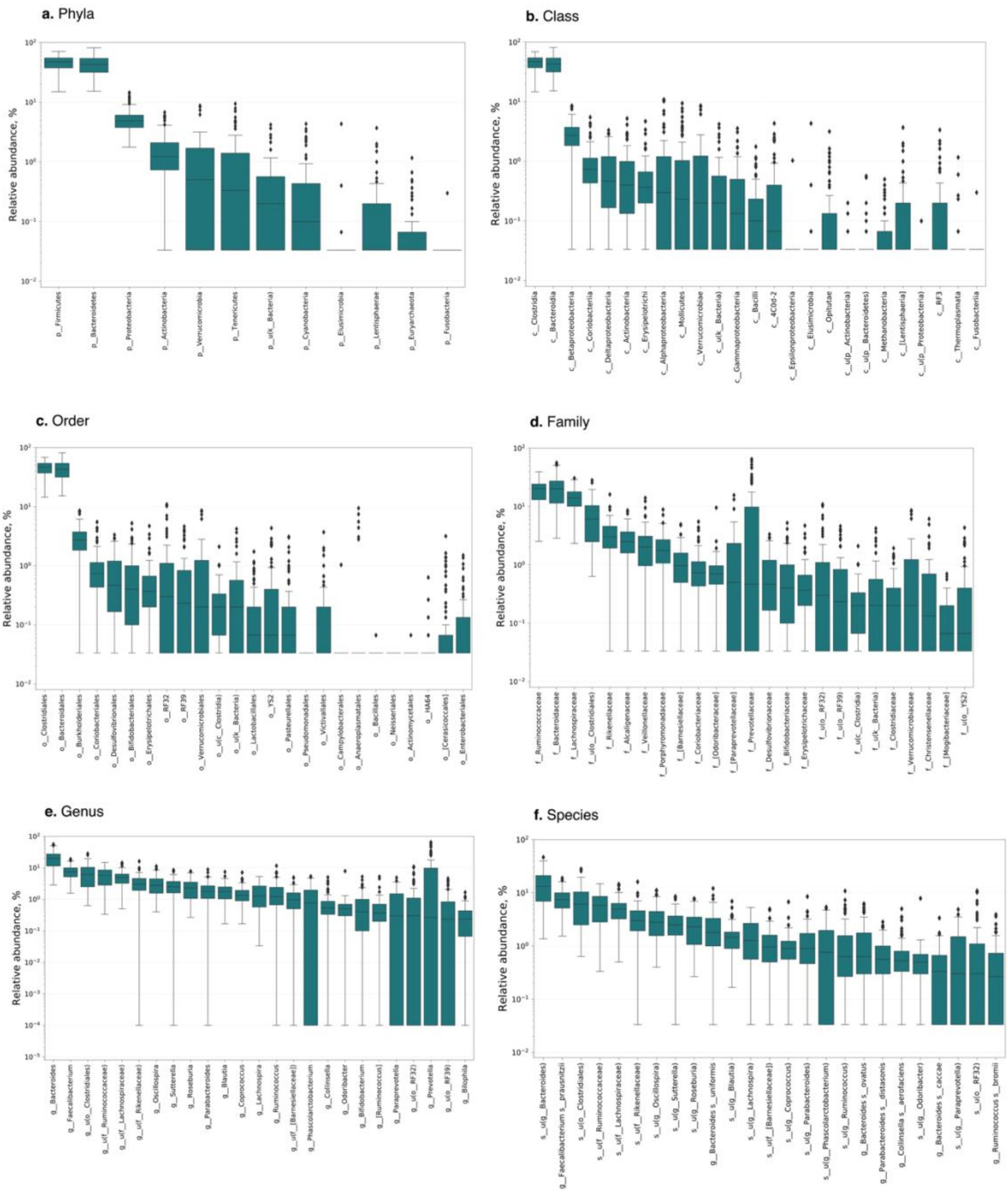

Figure A1. Boxplots representing the distribution of relative abundance for the 25 most abundant taxa in each taxonomic rank at baseline across all samples at baseline.

A2. Co-occurrence of microbial genera

Co-occurrence of microbial genera was analysed based on correlation analysis of their relative abundance using SPIEC-EASI software [166]. A diagrammatic representation of these data is provided in figure B1. Vertices showing genera and pairs of highly co-occurring genera are connected with grey lines. The graph shows the members of the cooperatives, i.e., groups of highly co-occurring genera corresponding to isolated components. Singleton vertices are omitted.

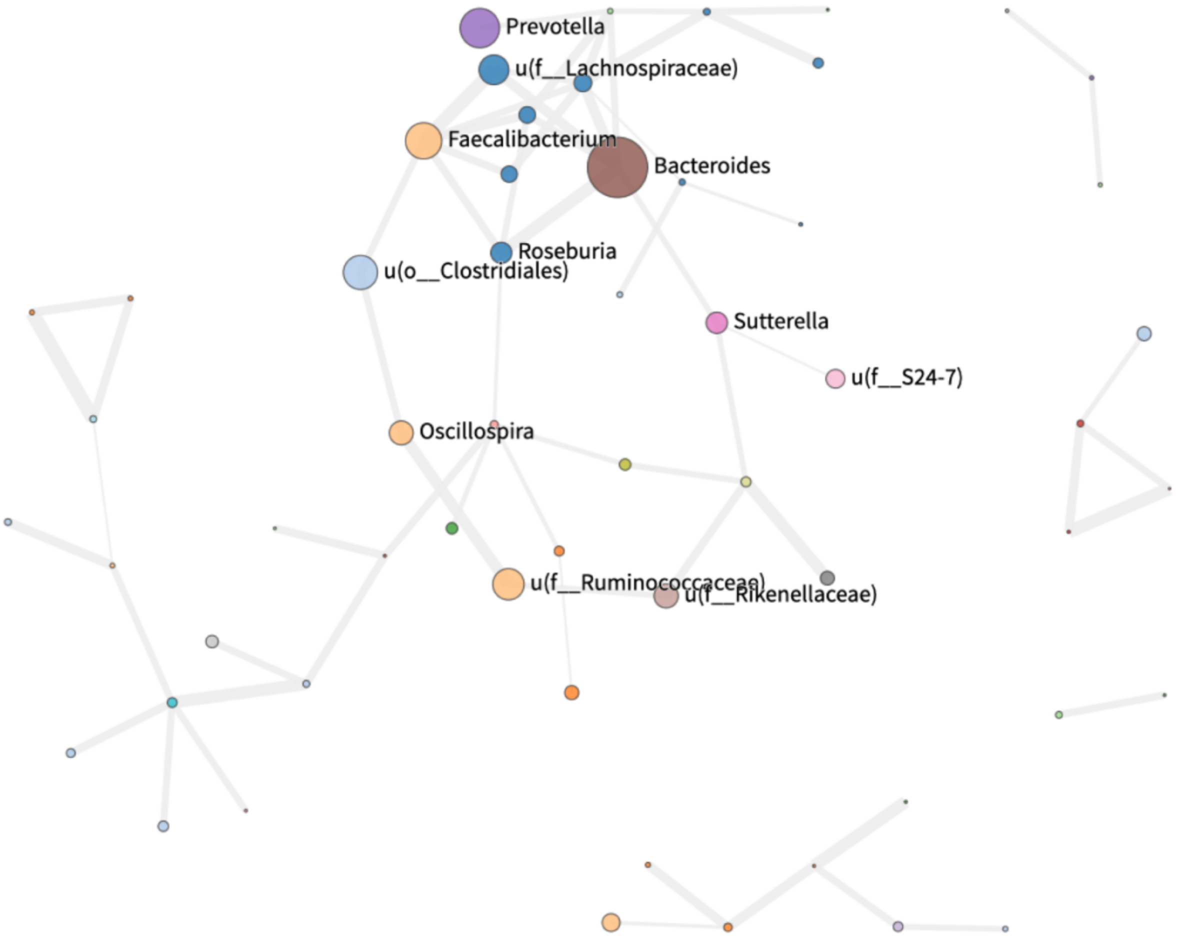

Figure A2. Parameters of SPIEC-EASI algorithm: Meinshausen and Bühlmann neighbourhood selection method [167], minimum lambda ratio= 0.1, number of lambda iterations = 20, model selection using StARS algorithm (number of StARS subsamples = 50). An interactive version of this figure is available online on http://bit.ly/ETH2021-0025a.

A3. Hierarchical clustering

The unrooted phylogenetic tree in figure B2 displays clustering of the samples by similarity of their taxonomic composition at varying levels of detail using Bray-Curtis as dissimilarity metric [168] and Ward's linkage method [169].

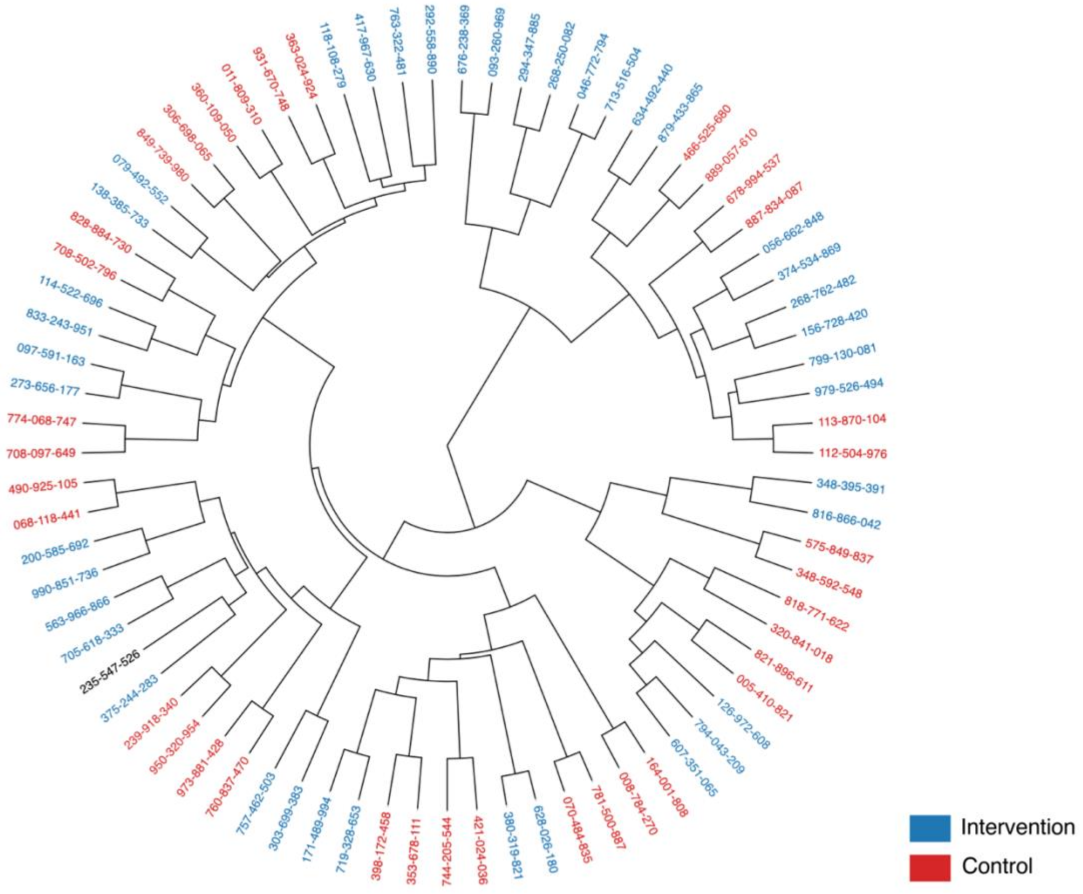

Figure A3. Hieararchical clustering of all study samples.

A4. Cladogram of differences

A tree-like summary of the taxa differentially abundant in the two groups, constructed using LefSe [170].

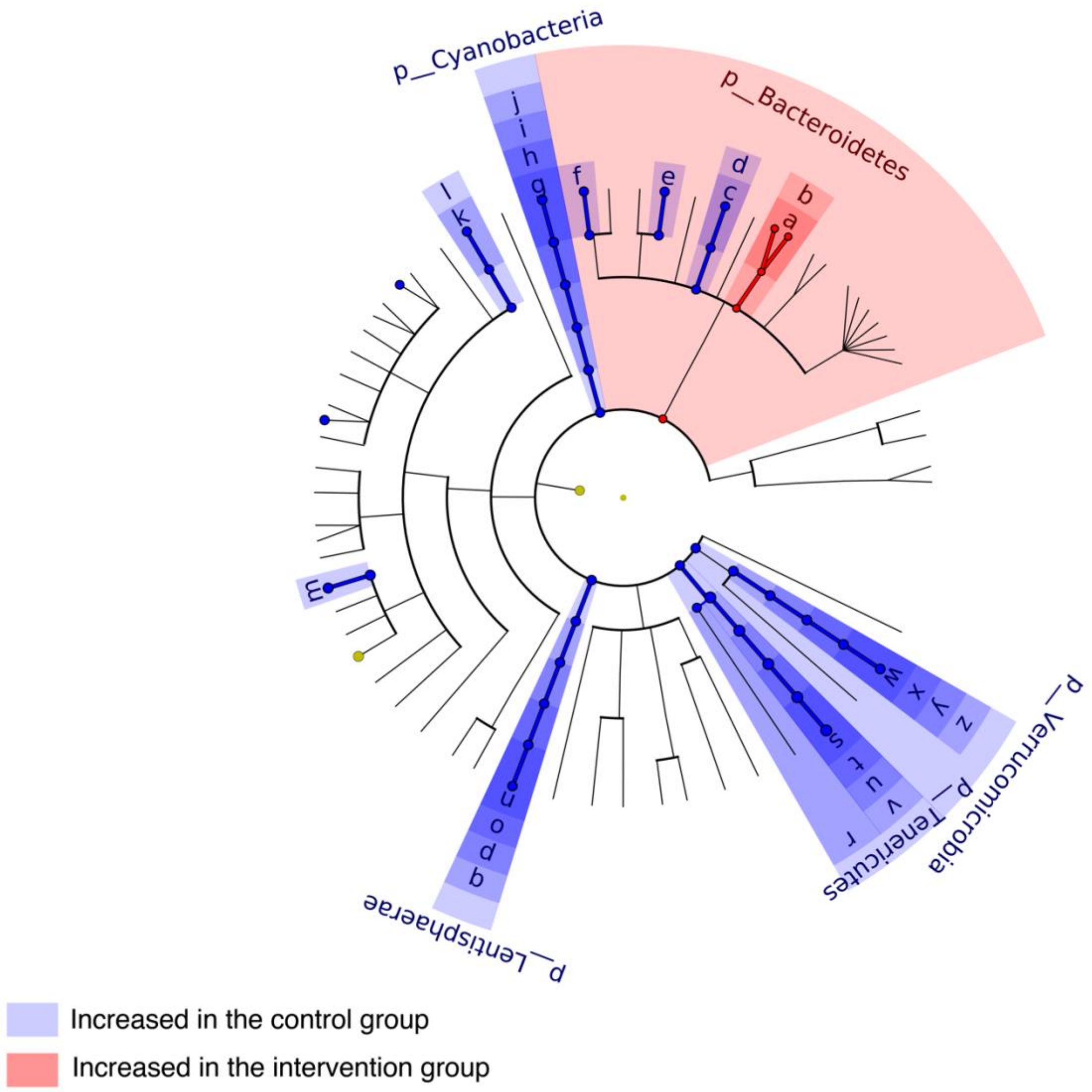

Figure A4. Cladogram of differences. The following denotations correspond to the taxa identified, where **p__** is for phylum; **o__** for order; **c__** for class; **f__** for family, and **g__** for genus.

a. g__Prevotella; **b.** f__Prevotellaceae **c.** g__u(f__S24-7); **d.** f__S24-7; **e.** g__Butyricimonas; **f.** g__[Prevotella]; **g.** g__u(o__YS2); **h.** f__u(o__YS2); **i.** o__YS2; **j.** c__4C0d-2; **k.** g__u(f__Christensenellaceae); **l.** f__Christensenellaceae; **m.** g__Acidaminococcus; **n.** g__u(f__Victivallaceae); **o.** f__Victivallaceae; **p.** o__Victivallales; **q.** c__[Lentisphaeria]; **r.** c__Mollicutes; **s.** g__u(o__ML615J-28); **t.** f__u(o__ML615J-28); **u.** o__ML615J-28; **v.** c__RF3; **w.** g__Akkermansia; **x.** f__Verrucomicrobiaceae; **y.** o__Verrucomicrobiales; **z.** c__Verrucomicrobiae
