# Supplementary material for: Impact of Plant-Based Meat Alternatives on the Gut Microbiota of Consumers: A Real-World Study
Source: Foods. 2021 Aug 30;10(9):2040. doi: 10.3390/foods10092040 (PMC8465665; doi:10.3390/foods10092040)
Supplement: Supplementary file 1 [file foods-10-02040-s001.zip › 12. MFPC059.pdf]

PRODUCT INFORMATION SHEET

MFPC059

MEATLESS FARM
LOVINGLY MADE FROM PLANTS

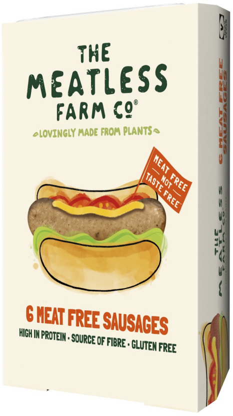

**BRAND**	Meatless Farm	
**PRODUCT DESCRIPTION**	Meat Free Sausages 6 x 50g	
**PRODUCT PACK SIZE**	300	
**PRODUCT EAN BARCODE**	5060626400429	
**Length mm**	184	
**Width mm**	130	
**Depth mm**	35	
**NET UNIT PACK WEIGHT (G)**<br>**Product content only**	300	
**GROSS UNIT PACK WEIGHT (G)**<br>**Includes product content + packaging**	324.4	
**UNITS PER CASE**	6	
**Length mm**	273	
**Width mm**	194	
**Depth mm**	120	
**NET CASE WEIGHT (G)**<br>**Product Content only**	1800	
**GROSS CASE WEIGHT (KG)**<br>**Includes product content + packaging**	2.09	
**CASE BARCODE**	15060626400426	
**PALLET TYPE**	EUR	GKN
**CASES ON PALLET LAYER (TI)**	16	20
**LAYERS ON PALLET (HI)**	14	14
**CASES ON PALLET (QTY)**	224	280
**UNIT PACKS ON PALLET (QTY)**	1344	1680
**NET PALLET WEIGHT (KG)**<br>**Product Content Only**	403.20	403.2
**GROSS PALLET WEIGHT (KG)**<br>**Includes product content + packaging and pallet weight**	649.15	808.19
**COMMODITY CODE**	21.06.102090	
**COUNTRY OF ORIGIN**	UK	
**TEMPERATURE BAND**	FROZEN	
**TOTAL PRODUCT LIFE DAYS**	365	

INGREDIENTS: Water, Pea Protein (18%), Vegetable Oils (Rapeseed, Shea, Sunflower), Thickener: E461, Coconut Oil, Spices and Herbs, Pea Fibre, Onion Powder, Potato Starch, Potato Fibre, Rice Protein, Natural Flavouring, Salt, Acidity Regulator: E300, Vegetable and Fruit Extracts (Beetroot, Radish, Tomato), Yeast Extract, Caramelised Carrot Concentrate, Carrot Concentrate. Sausages filled into sodium alginate casings.

ALLERGENS: **No allergens**

STORAGE: For Best Before, see top of pack. Keep frozen. Do not exceed use by date. Defrost thoroughly (in a refrigerator) before use. Once defrosted use within 24 hours. Do not re-freeze.

COOKING INSTRUCTIONS: Pre-heat grill to a medium heat. Remove from packaging. Place the sausages onto the wire rack of a grill tray. Cook for 5-7 minutes turning frequently, reduce heat if necessary. Ensure food is piping hot, serve and enjoy!

NUTRITIONAL INFORMATION PER 100G

KJ	974
Kcal	234
Fat	15.9g
of which saturated	5.0g
Carbohydrates	6.9g
of which sugars	0.3g
Fibre	3.2g
Protein	14.4g
Salt	1.27g

*Analytical results

SALES CONTACTS:
