# Supplementary material for: Impact of Plant-Based Meat Alternatives on the Gut Microbiota of Consumers: A Real-World Study
Source: Foods. 2021 Aug 30;10(9):2040. doi: 10.3390/foods10092040 (PMC8465665; doi:10.3390/foods10092040)
Supplement: Supplementary file 1 [file foods-10-02040-s001.zip › 11. MFPC057.pdf]

PRODUCT INFORMATION SHEET

MFPC057

MEATLESS FARM
LOVINGLY MADE FROM PLANTS

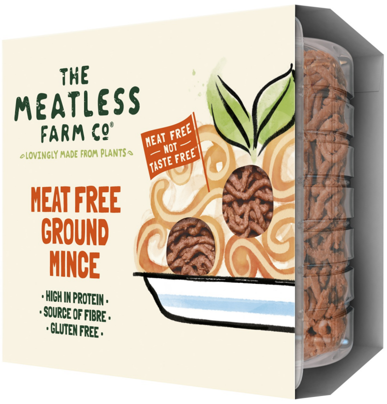

**BRAND**	Meatless Farm	
**PRODUCT DESCRIPTION**	Meat Free Ground Mince 400g	
**PRODUCT PACK SIZE**	400	
**PRODUCT EAN BARCODE**	5060626400405	
**Length mm**	154	
**Width mm**	196	
**Depth mm**	55	
**NET UNIT PACK WEIGHT (G)**<br>**Product content only**	400	
**GROSS UNIT PACK WEIGHT (G)**<br>**Includes product content + packaging**	431	
**UNITS PER CASE**	6	
**Length mm**	470	
**Width mm**	220	
**Depth mm**	128	
**NET CASE WEIGHT (G)**<br>**Product Content only**	2400	
**GROSS CASE WEIGHT (KG)**<br>**Includes product content + packaging**	2.81	
**CASE BARCODE**	15060626400402	
**PALLET TYPE**	EUR	GKN
**CASES ON PALLET LAYER (TI)**	7	10
**LAYERS ON PALLET (HI)**	13	12
**CASES ON PALLET (QTY)**	91	120
**UNIT PACKS ON PALLET (QTY)**	546	720
**NET PALLET WEIGHT (KG)**<br>**Product Content Only**	218.4	218.4
**GROSS PALLET WEIGHT (KG)**<br>**Includes product content + packaging and pallet weight**	383.18	500.32
**COMMODITY CODE**	21.06.102090	
**COUNTRY OF ORIGIN**	UK	
**TEMPERATURE BAND**	FROZEN	
**TOTAL PRODUCT LIFE DAYS**	365	

INGREDIENTS: Water, Soya Protein Concentrate (19%), Rapeseed Oil, Soya Protein Isolate (5%), Pea Protein (4.5%), Chicory Root Fibre, Thickener: Methyl Cellulose, Natural Flavouring, Caramelised Carrot Concentrate, Carrot Fibre, Rice Protein, Yeast Extracts, Vegetable and Fruit Extracts (Beetroot, Radish, Tomato), Carrot Concentrate, Emulsifier: Soya Lecithin, Acidity Regulator: Ascorbic Acid, Salt, Vitamins and Minerals (Niacin, Zinc, Iron, Vitamin B6, Vitamin B2, Vitamin B1, Vitamin B12)

ALLERGENS: **Soya**

STORAGE: For Use By, see top of pack. Keep frozen. Do not exceed use by date. Defrost thoroughly (in a refrigerator) before use. Once defrosted use within 24 hours. Do not re-freeze.

COOKING INSTRUCTIONS: For best results use mince directly from the refrigerator. Remove all packaging. Heat a non stick pan on medium high heat, add some oil, gently flake the mince into the pan and don't touch for one minute. Continue cooking for 12-15 minutes stirring often. Add more oil if desired. Finish preparing your favourite dish as required, serve and enjoy!

NUTRITIONAL INFORMATION PER 100G

KJ	831
Kcal	199
Fat	10.9g
of which saturated	3.9g
Carbohydrates	7.8g
of which sugars	0.1g
Fibre	4.9g
Protein	19.1g
Salt	0.62g

*Analytical results

SALES CONTACTS:
