# Supplementary material for: Impact of Plant-Based Meat Alternatives on the Gut Microbiota of Consumers: A Real-World Study
Source: Foods. 2021 Aug 30;10(9):2040. doi: 10.3390/foods10092040 (PMC8465665; doi:10.3390/foods10092040)
Supplement: Supplementary file 1 [file foods-10-02040-s001.zip › 10. MFPC044.pdf]

Customer Specification

The Meatless Farm Co, Graphical House, 2 Wharf Street, Leeds, LS2 7EQ
www.meatlessfarm.com

**Product Name:**	Meat Free Meatballs		**Barcode:**	5060626400351	
**Description:**	Frozen Meat Free Meatballs	**Recipe Code:**	MFR071S	**MF Code:**	MFPC044

**1.0 Product Information**	
**1.1 Product Title**	
Meat Free Meatballs	
**1.2 Sub-Title**	
Meat Free Not Taste Free!	
**1.3 Legal Title**	
Meat free meatballs made with pea protein	
**1.4 Ingredient Declaration (Allergens in Bold)**	
Water, Pea Protein, Rapeseed Oil, Potato Starch, Thickener: Methyl Cellulose, Dried Onion, Pea Fibre, Garlic, Caramelised Carrot Concentrate, Yeast Extracts, Salt, Potato Fibre, Vegetable and Fruit Extracts (Beetroot, Radish, Tomato), Natural Flavouring, Ground Black Pepper, Carrot Concentrate, Dried Parsley, Acidity Regulator: Ascorbic Acid	
**1.5 Declared Weight**	945g ℮<br>Approx 63 x 15g per bag
**1.6 Number of Servings**	Approx 3-4 meatballs per serving
**1.7 Serving size (in weight)**	Approx 15-16 servings
**1.8 County of Manufacture Statement**	MADE IN BRITAIN
**1.9 Company Address Statement**	The Meatless Farm Co, Graphical House, 2 Wharf Street, Leeds, LS2 7EQ<br>info@meatlessfarm.com

2.0 Allergen, Intolerance and Additives Information		
**2.1 Allergens**		
**Component**	**Present? (Y/N)**	**Ingredient Present In**
**Peanut** or its derivatives	N	
**Tree nut** or derivatives (Please state which in details)	N	
**Sesame** or its derivatives	N	
**Milk** or its derivatives	N	
**Egg** or its derivatives	N	
**Fish** or its derivatives	N	
**Crustaceans (Shellfish)** or its derivatives	N	
**Molluscs** or its derivatives	N	
**Soya** or its derivatives	N	
**Sulphites** at concentrations >10ppm	N	
**Celery** or its derivatives	N	
**Mustard** or its derivatives	N	
**Lupin** or its derivatives	N	
**Gluten** at concentrations >20ppm (Please state which in details)	N	

2.2 Intolerance Information					
	Yes	No		Yes	No
**Free from Colours (natural)**	X		**Free from Butylated Hydroxyanisole (BHA) and Butylated Hydroxytoluene (BHT)**	X	
**Free from Colours (artificial)**	X		**Free from Hydrolysed Vegetable Protein**	X	
**Free from Flavourings (natural)**		X	**Free from Hydrogenated Fat**	X	
**Free from Flavourings (artificial)**	X		**Free from Added Sugar**	X	
**Free from Preservatives**	X		**Free from Added Salt**		X
**Free from Additives**		X	**Free from all animal products**	X	
**Free from Sweeteners**	X		**Free from Caffeine**	X	
**Free from Benzoates**	X		**Free from Potassium based salt substitute**	X	
**Free from Glutamates (including MSG)**	X		**Free from Yeast**		X
**Free from Aspartame**	X		**Free from AZO and Coal Tar Dyes**	X	
**Free from Phenylalanine**	X		**Free from Irradiated ingredients**	X	
**Free from Garlic**		X	**Free from Maize or Maize derivatives**	X	
**Free from GMO ingredients**	X				

2.3 Suitability Information					
	Yes	No		Yes	No
**Ovo-lacto Vegetarians**	X		**Nut/Seed Allergy Sufferers**	X	
**Vegans**	X		**Soya Allergy Sufferers**	X	
**Coeliacs**	X		**Organic products**		X
**Egg Allergy Sufferers**	X		**Halal products**		X*
**Milk Allergy Sufferers**	X		**Kosher products**		X
			***NOT CERTIFIED**		

3.0 Nutritional Information					
**3.1 Back of Pack Nutrition** (last 3 columns do not need to appear on back of pack)					
Test	PER 100G (AS SOLD)	PER 100 (AS SOLD)	Calculated/ Analytical?	RI %	RDI
**Energy kJ**	929	929	Analytical		8400
**Energy kcal**	223	223	Analytical		2000
**Total Fat**	11.9	11.9	Analytical		70
**Of which saturates**	0.8	0.8	Analytical		20
**Carbohydrate**	11.8	11.8	Analytical		-
**Of which sugars**	0.9	0.9	Analytical		90
**Fibre (by AOAC method)**	4.2	4.2	Analytical		-
**Protein**	15.0	15.0	Analytical		-
**Sodium (mg)**	544	544	Analytical		-
**Salt**	1.36	1.36	Analytical		6
**VITAMINS:**					
**Niacin**	N/A	N/A	N/A		16mg
**Vitamin B6**	N/A	N/A	N/A		1.4mg
**Vitamin B2 (Riboflavin)**	N/A	N/A	N/A		1.4mg
**Vitamin B1 (Thiamin)**	N/A	N/A	N/A		1.1mg
**Vitamin B12**	N/A	N/A	N/A		2.5µg
**MINERALS**	N/A	N/A	N/A		
**Zinc**	N/A	N/A	N/A		10mg
**Iron**	N/A	N/A	N/A		14mg

3.2 Front of Pack Nutrition	
**Type e.g. Energy Only or Full front of Pack**	N/A
**Statement to appear e.g. per 50g serving, per burger**	N/A
**Other**	N/A

4.0 Preparation and Cooking Instructions			
**Method e.g. Hob, Microwave, Oven, Grill**	**Time**	**Temperature to Include wattage, degrees, fan and gas if application**	**Special Instructions**
Hob	8 – 10 minutes	Medium Heat	N/A

4.1 Recommended Cooking Instructions

Remove from all packaging. Heat a little oil in a saucepan over a medium heat. Cook over a medium heat for 8 to 10 minutes turning occasionally.

5.0 Shelf Life and Storage Conditions

5.1 Shelf Life and Storage

**Storage**		**Shelf Life**	
Maximum Delivery Temperature:	-18°C	Total shelf-life from production:	12 months – TBC shelf life testing ongoing
Storage Temperature	-18°C	Customer Shelf Life	9 months – TBC shelf life testing ongoing
Special Storage Conditions	N/A	Shelf-life once opened	N/A
Suitable for Freezing:	Product is frozen	Shelf-life once defrosted (if applicable)	48 hours
Recommended Defrost Conditions (if applicable)	Defrost in chilled temperatures	Freeze Thaw Stable Shelf Life:	N/A

5.2 Recommended Storage Instructions

Keep frozen. Do not exceed use by. Defrost thoroughly (in a refrigerator) before use for a minimum of 48 hours. Once defrosted use within 48 hours. Do not re-freeze.

6.0 Claims and Logos

6.1 Claims

Claim	Location
N/A	

6.2 Logos

Logo	Location

N/A	

6.3 Recycling	
**Statement**	**Location**
N/A	

6.4 Other	
E.g. facts, social media links	
**Other**	**Location**
N/A	

7.0 Product Standards	
**7.1 Photo Standards**	
**Green**	**Red**
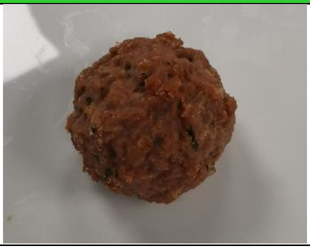	

7.2 Organoleptic Standards		
	**Green**	**Red**
**Appearance**	Beige with a hint of pink colour. Uniform & Round Edges May Be a Little Rustic with Small Fragments Missing. Specks of green herbs and black pepper	Tear drop shape, squished. Missing herbs or black pepper.
**Aroma**	Herby savoury aroma	No Herb Aroma, Any off Aroma's
**Flavour**	N/A	N/A
**Texture**	Firm & Coarse Textured with White Flecks of Fat Evident	Reform if Too Soft/Pappy

THE MEATLESS FARM Co LOVINGLY MADE FROM PLANTS

Customer Specification

The Meatless Farm Co, Graphical House, 2 Wharf Street, Leeds, LS2 7EQ
www.meatlessfarm.com

THE MEATLESS FARM Co LOVINGLY MADE FROM PLANTS

7.3 Microbiology						
**Test**	**Method**	**Frequency**	**Unit**	**Target**	**Report**	**Reject**
TVC	TBC	Each Prodn	cfu/g	<1X105	N/A	>1X106
E Coli	TBX or similar	Each Prodn	cfu/g	<10	N/A	> 50
Staph Aureus	BP	Each Prodn	cfu/g	<1X102	N/A	>2X102
Yeasts Moulds	DRBCA	Each Prodn	cfu/g	<1X102	N/A	>2X102
Coliforms	TBC	Each Prodn	cfu/g	<1.0X103	N/A	>5.0X103
Clostridia (Sulphite-reducing)	TBC	Each Prodn	cfu/g	<50	N/A	>1X102
Salmonella spp	ISO 6579-1:2017	Monthly	cfu/g	Abs 25g	N/A	Present
Listeria spp	ISO 11290:1998 + Amd1:2004	Monthly	cfu/g	Abs 25g	N/A	Present

7.4 Chemical Tests						
E.g. aw, pH						
**Test**	**Method**	**Frequency**	**Unit**	**Target**	**Min**	**Max**
N/A						

7.5 Quality Control and Production Checks				
**Test**	**Frequency**	**Standard**	**Tolerance**	**Action if outside specification**
**Metal Detector**	Start-up, every 30 minutes and end of production. Piece sizes: 4mm Non Ferrous, 4mm Ferrous, 5mm Stainless Steel	3mm Fe, 4mm non-Fe & 5mm Stainless Steel	No metal detected	Reject bags detected
**Sieving**	N/A	N/A	N/A	N/A
**Glass & Hard plastic**	Daily production glass and hard plastic checks,	N/A	N/A	N/A

	daily production pre-start checks.			
**Weights**	Start-up & every 15 minutes.	Raw Meatballs 15.5g ± 2g<br>Cooked Meatballs 15g± 2 g<br><br>Final Weight 945g	Raw and Cooked ± 2g	Reset machinery to ensure meatball weight is achieved
**Coding**	Beginning, middle and end of production.	As per agreed spec.	As per agreed spec.	Quarantine & re-code as necessary

**8.0 Product Packaging**							
**8.1 Level**							
**Level**	**Description**	**Material & Thickness**	**Colour**	**Dimensions**	**Weight**	**Sealing Method**	**Recyclable?**
Primary	Bag	Blue Food Grade Bag	Blue	320 x 320mm	14g	Heat Sealed	N
Secondary	Box	B Flute Card Case	Tan	458 x 203 x 118mm	204g	Tape	Y
Tertiary	Pallet wrap	PE, 50 µm	Blue tint	N/A	N/A	N/A	N

**8.2 Configuration**	
**Packed to Average or Minimum Weight:**	Averge
**Weight of Unit Supplied:**<br>**T1:**<br>**T2:**	945g ℮<br>N/A<br>N/A
**Number of Units per SRP/Outer Case:**	4
**Number of Cases per Pallet:**	120 – 10/layer, 12 layers hgh
**Type of Pallet:**	Wood 4-way entry Chep or similar (1200 x 1000 mm)
**Pallet Height:**	1.63m

**8.3 Gas Flushing**	
**Gas**	**Usage %**
**Carbon Dioxide**	N/A
**Nitrogen**	N/A
**Oxygen**	N/A
**Argon**	N/A
**Carbon Monoxide**	N/A
**Vacuum**	N/A

Customer Specification

The Meatless Farm Co, Graphical House, 2 Wharf Street, Leeds, LS2 7EQ
www.meatlessfarm.com

Specification Review			
**Version**	**Date**	**Amended By**	**Details of Amendments**
1	27.11.19	S Lewis	New Specification
2	30.01.2020	S Lewis	Updated suitability information
3	27.02.2020	S Lewis	Updated allergen information and suitability to May Contain Soya
4	11.03.2020	S Lewis	Removed alibi statement and updated suitability information
5	23.07.2020	S Leighton	Not Kosher Suitable

Warranty

This product is manufactured according to the following (but not limited to) the relevant UK food legislation, the Food Safety Act (1990 and all relevant amendments and regulations) and recognised good manufacturing practises, the Materials and Articles in Contact with Food Regulations (1987 and all relevant amendments and regulations)

This specification contains confidential information and is the sole property of The Meatless Farm Co.

Safety Information may still appear TBC until all data is gathered. This does not affect finished product.

Signegd	
**Name**	Sarah Leighton
**Position**	Senior Specification Technologist
**Signature**	S.Leighton
**Date**	23.07.2020
