# Supplementary material for: Impact of Plant-Based Meat Alternatives on the Gut Microbiota of Consumers: A Real-World Study
Source: Foods. 2021 Aug 30;10(9):2040. doi: 10.3390/foods10092040 (PMC8465665; doi:10.3390/foods10092040)
Supplement: Supplementary file 1 [file foods-10-02040-s001.zip › 9. MFPC037.pdf]

Customer Specification

The Meatless Farm Co, Graphical House, 2 Wharf Street, Leeds, LS2 7EQ
www.meatlessfarm.com

**Product Name:**	4 Meat Free Sausage Patties		**Barcode:**	860001533651	
**Description:**	Meat Free Sausage Patties 240g Freeze Thaw	**Recipe Code:**	MFR107C	**MF Code:**	MFPC037

**1.0 Product Information**	
**1.1 Product Title**	
4 MEAT FREE SAUSAGE PATTIES	
**1.2 Sub-Title**	
MEAT FREE NOT TASTE FREE™	
**1.3 Legal Title**	
meat free sausage patties made with pea protein	
**Ingredient Declaration & Allergens**	
Water, Pea Protein, Canola Oil, Shea Oil, Methylcellulose, Less than 2% of: Seasoning (Spices (White Pepper, Nutmeg, Coriander), Onion Powder, Sage, Ascorbic Acid (Antioxidant), Sunflower Oil), Coconut Oil, Pea Fiber, Potato Fiber, Natural Flavor, Salt, Rice Protein, Vegetable and Fruit Extracts for Color (Beetroot, Radish, Tomato), Yeast Extract, Ascorbic Acid (Antioxidant), Caramelized Carrot Concentrate, Sage, Coriander, Nutmeg, Carrot Concentrate<br>CONTAINS: COCONUT, SHEANUT	
**1.5 Declared Weight – Net weight in lbs & oz and g in brackets**	NET WT. 8.46OZ (240G)
**1.6 Number of Servings**	2 per container
**1.7 Serving size (in weight)**	2 Patties (120g)
**1.8 County of Manufacture Statement**	PRODUCT OF THE UK.
**1.9 Company Address Statement**	Meatless Farm North America<br>71 McMurray Rd. Suite 104<br>Pittsburgh, PA 15241<br>usa-info@meatlessfarm.com

2.0 Allergen, Intolerance and Additives Information		
**3.1 Allergens**		
**Component**	**Present? (Y/N)**	**Ingredient Present In**
**Peanut**	N	
**Tree nut**	Y	Sheanut, Coconut
**Milk**	N	
**Egg**	N	
**Fish**	N	
**Crustaceans (Shellfish)**	N	
**Soyabeans**	N	
**Wheat**	N	

2.2 Intolerance Information					
	Yes	No		Yes	No
**Free from Colours (natural)**		X	**Free from Butylated Hydroxy anisole (BHA) and Butylated Hydroxytoluene (BHT)**	X	
**Free from Colours (artificial)**	X		**Free from Hydrolysed Vegetable Protein**	X	
**Free from Flavourings (natural)**		X	**Free from Hydrogenated Fat**	X	
**Free from Flavourings (artificial)**	X		**Free from Added Sugar**	X	
**Free from Preservatives**	X		**Free from Added Salt**		X
**Free from Additives**		X	**Free from all animal products**	X	
**Free from Sweeteners**	X		**Free from Caffeine**	X	
**Free from Benzoates**	X		**Free from Potassium based salt substitute**	X	
**Free from Glutamates (including MSG)**	X		**Free from Yeast**		X
**Free from Aspartame**	X		**Free from AZO and Coal Tar Dyes**	X	
**Free from Phenylalanine**	X		**Free from Irradiated ingredients**	X	
**Free from Garlic**	X		**Free from Maize or Maize derivatives**	X	
**Free from GMO ingredients**	X				

2.3 Suitability Information					
	Yes	No		Yes	No
**Ovo-lacto Vegetarians**	X		**Nut/Seed Allergy Sufferers**		X
**Vegans**	X		**Soya Allergy Sufferers**	X	
**Coeliacs**	X		**Organic products**		X
**Egg Allergy Sufferers**	X		**Halal products**		X*
**Milk Allergy Sufferers**	X		**Kosher products**	X	
			***NOT CERTIFIED**		

3.0 Nutritional Information	
**3.1 Nutrition Facts**	
**Nutrition to appear on back of pack:**	**Per serving and per container**
**Serving Information to appear in nutrition panel:**	A2 servings per container<br>Serving Size: 2 Patties 120g
**Daily Value Information to appear under nutrition:**	*The % Daily Value (DV) tells you how much a nutrient in a serving of food contributes to a daily diet. 2,000 calories a day is used for general nutrition advice

Test	Per Serving	Per container	Calculated/ Analytical?	% Daily Value	Daily Value
**Calories**	280	560	Analytical		-
**Total Fat**	18g	35g	Analytical	22/45	65g
Saturated Fat	5g	11g	Analytical	25/51	20g
Trans Fat	0g	0g	Analytical		
**Cholesterol**	0mg	0mg	Analytical	0/0	300mg
**Sodium (mg)**	610mg	1210mg	Analytical	26/52	2400mg
**Total Carbohydrate**	12g	23g	Analytical	4/8	300g
Dietary Fiber	3g	5g	Analytical	9/17	25g
Total Sugars	0g	0g	Analytical		-
Includes xg Added Sugars	0g	0g	Analytical	0/0	-
**Protein**	20g	41g	Analytical	15/61	50g
**Vitamin D**	0mcg	0mcg	Analytical	0/0	400IU
**Calcium**	60mg	110mg	Analytical	4/8	1000mg
**Iron**	5mg	10mg	Analytical	25/50	18mg
**Potassium**	190mg	372mg	Analytical	4/8	3500mg
**Thiamine**	N/A	N/A	N/A	N/A	1.5mg
**Riboflavin**	N/A	N/A	N/A	N/A	1.7mg
**Niacin**	N/A	N/A	N/A	N/A	20mg
**Vitamin B6**	N/A	N/A	N/A	N/A	2mg
**Vitamin B12**	N/A	N/A	N/A	N/A	6ug
**Zinc**	N/A	N/A	N/A	N/A	15mg

4.0 Preparation and Cooking Instructions			
**Method e.g. Hob, Microwave, Oven, Grill**	**Time**	**Temperature to Include wattage, degrees, fan and gas if application**	**Special Instructions**
Frying	6 minutes	High	Couple of tablespoons of oil

4.1 Recommended Cooking Instructions
Cook from thawed. Remove all packaging. Heat a couple of tablespoons of oil in a non-stick pan on a high heat. Add your sausage patties and cook for approximately 6 minutes turning occasionally, reduce heat if necessary. Ensure food is piping hot, serve and enjoy!

5.0 Shelf Life and Storage Conditions			
**5.1 Shelf Life and Storage**			
**Storage**		**Shelf Life**	
Maximum Delivery Temperature:	<-18°C	Total shelf-life from production:	Frozen – 12 months
Storage Temperature	Frozen <-18°C<br>Sold Chilled 3°C ± 2°C	Customer Shelf Life	Frozen - 9 Months
Special Storage Conditions	N/A	Shelf-life once opened	Use within 24 hours
Suitable for Freezing:	Product is frozen and not suitable for re-freezing	Shelf-life once defrosted (if applicable)	Once defrosted – 10 days
Recommended Defrost Conditions (if applicable)	Defrost in chilled temperatures.	Freeze Thaw Stable Shelf Life:	Once defrosted – 10 Days
**5.2 Recommended Storage Instructions**			
STORAGE: If purchased thawed, see use by on top of pack. Keep refrigerated, once opened consume within 24 hours and do not exceed the use by date. Not suitable for re-freezing. If purchased frozen, see use by printed on film. Keep frozen and do not exceed the use by date. Thaw in a refrigerator before use, once thawed, cook & consume within 10 days.			

6.0 Claims and Logos	
**6.1 Claims**	
**Claim**	**Location**
GOOD SOURCE OF PROTEIN	Front of Pack
NO SOY	Front of Pack
GLUTEN FREE	Front of Pack
100% VEGAN	Back of Pack
Kosher	Back of Pack

6.2 Logos	
**Logo**	**Location**
THE MEATLESS FARM CO<br>LOVINGLY MADE FROM PLANTS®	Front, Side and Back of Pack
New Flash	Front of Pack

6.3 Recycling	
**Statement**	**Location**
Sleeve and tray are both widely recycled.	Back of Pack

6.4 Other	
E.g. facts, social media links	
**Other**	**Location**
HERE AT THE MEATLESS FARM CO. WE HAVE CREATED A DELICIOUS PLANT BASED ALTERNATIVE WITH GREAT TASTE AND TEXTURE!	Side of Pack
Plant-based made easy	
www.meatlessfarm.com<br>Facebook logo<br>Instagram logo<br>Twitter logo	Side of Pack
Copyright © 2020 The Meatless Farm Ltd. All Rights Reserved.	Back of Pack
MFPC037_V2	Back of Pack
Keep Refrigerated<br>If Purchased Refrigerated, Use By:<br>Retailer: Apply Refrigerated "Use By" Date	Front of Pack

7.0 Product Standards

7.1 Photo Standards

Green	Red
**Raw:**<br>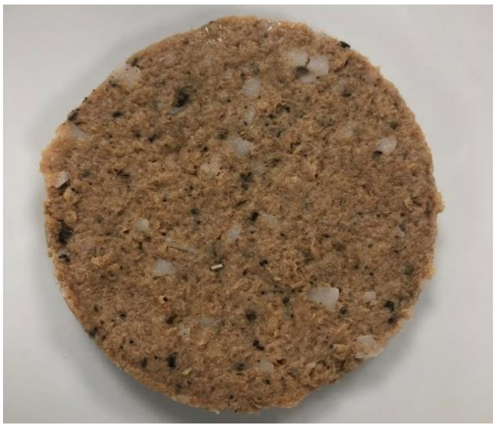	**Raw:**<br>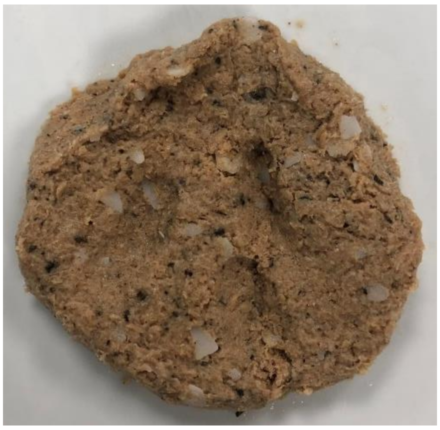

7.2 Organoleptic Standards

**Appearance**	Beige with a hint of pink colour. Uniform & Round Edges May Be a Little Rustic with Small Fragments Missing. Specks of green herbs and black pepper	Oval/Egg Shaped, No Missing Edges>10mm.<br>Missing herbs or black pepper.
**Aroma**	Herby savoury aroma	No Herb Aroma, Any off Aroma's
**Flavour**	N/A	N/A
**Texture**	Firm & Coarse Textured with White Flecks of Fat Evident	Reform if Too Soft/Pappy

**7.3 Microbiology**						
**Test**	**Method**	**Frequency**	**Unit**	**Target**	**Report**	**Reject**
TVC	PCA	Each Prodn	cfu/g	<100000	N/A	>50000000
Enteros	VRBA	Each Prodn	cfu/g	<1000	N/A	>100000
E Coli	TBX or similar	Each Prodn	cfu/g	<100	N/A	>1000
Staph Aureus	BP	Each Prodn	cfu/g	<20	N/A	>20
Yeasts Moulds	DRBCA	Each Prodn	cfu/g	<1000	N/A	>100 000
Coliforms	TBC	Each Prodn	cfu/g	<1000	N/A	>5000
LAB	ISO15214:1998	Each Prodn	cfu/g	<10 000	N/A	>50 000 000
Clostridia (sulfite-reducing)	TBC	Each Prodn	cfu/g	<100	N/A	>100
Salmonella spp	ISO 6579-1:2017	Monthly	cfu/g	Abs 25g	N/A	Present
Listeria spp	ISO 11290:1998 + Amd1:2004	Monthly	cfu/g	Abs 25g	N/A	Present

**7.4 Chemical Tests**						
E.g. aw, pH						
**Test**	**Method**	**Frequency**	**Unit**	**Target**	**Min**	**Max**
N/A						

**7.5 Quality Control and Production Checks**				
**Test**	**Frequency**	**Standard**	**Tolerance**	**Action if outside specification**

THE MEATLESS FARM Co LOVINGLY MADE FROM PLANTS

**Metal Detector**	Start-up, every 30 minutes and end of production	3mm Fe, 4mm non-Fe & 5mm Stainless Steel	No metal detected	Reject product detected
**Sieving**	N/A	N/A	N/A	N/A
**Glass & Hard plastic**	Daily production glass and hard plastic checks, daily production pre-start checks.	N/A	N/A	N/A
**Weights**	Off-line checkweighing – 5 packs ever 15-20 minutes	≥T1	Reject packs below T1 in initial stages	Quarantine back to last successful check & re-weigh/adjust as necessary.
**Coding**	Checked at start-up	As per agreed spec.	As per agreed spec.	Quarantine & re-code as necessary

8.0 Product Packaging

8.1 Level

**Level**	**Description**	**Material & Thickness**	**Colour**	**Dimensions**	**Weight**	**Sealing Method**	**Recyclable?**
Primary	Sleeve	r/PET SUKANO	Printed	128 x 33 x 155mm	Approx. 12.5g	N/A	Yes
Primary	Tray	r/PET SUKANO	Clear	127 x 30 x 219	Approx. 13g	N/A	Yes
Primary	Film	TBC	Clear	132 x 224	Approx. 3g	Heat Sealed	No
Secondary	Outercase	150WK T B	Printed	273 x 194 x 120mm	Approx 140g	Clear Tape	Yes

8.2 Configuration

**Packed to Average or Minimum Weight:**	Average
**Weight of Unit Supplied:**<br>**T1:**<br>**T2:**	240g (8.46oz)<br>231g<br>222g
**Number of Units per SRP:**	6 Packs per case
**Number of Cases per Pallet:**	18 cases/layer; 13 layers/pallet: 234 cases/pallet
**Type of Pallet:**	Wood 4-way entry Chep or similar (1200 x 1000 mm)
**Pallet Height:**	1.63m

8.3 Gas Flushing	
**Gas**	**Usage %**
**Carbon Dioxide**	30%
**Nitrogen**	N/A
**Oxygen**	70%
**Argon**	N/A
**Carbon Monoxide**	N/A
**Vacuum**	N/A
**Testing**	Start-up, every 30 mins and at end of production using a calibrated gas analyser.

Specification Review			
**Version**	**Date**	**Amended By**	**Details of Amendments**
1	27.02.2020	S Lewis	New specification
2	12.06.2020	S Leighton	Update to cooking instructions & storage

Warranty
This product is manufactured according to the following (but not limited to) the relevant UK food legislation, the Food Safety Act (1990 and all relevant amendments and regulations) and recognised good manufacturing practises, the Materials and Articles in Contact with Food Regulations (1987 and all relevant amendments and regulations)<br><br>This specification contains confidential information and is the sole property of The Meatless Farm Co.<br><br>Safety Information may still appear TBC until all data is gathered. This does not affect finished product.

Signed	
**Name**	Sarah Leighton
**Position**	Senior Specification Technologist
**Signature**	S.Leighton
**Date**	12.06.2020
