# Supplementary material for: Impact of Plant-Based Meat Alternatives on the Gut Microbiota of Consumers: A Real-World Study
Source: Foods. 2021 Aug 30;10(9):2040. doi: 10.3390/foods10092040 (PMC8465665; doi:10.3390/foods10092040)
Supplement: Supplementary file 1 [file foods-10-02040-s001.zip › 8. MFPC058.pdf]

PRODUCT INFORMATION SHEET

MFPC058

MEATLESS FARM
LOVINGLY MADE FROM PLANTS

**BRAND**	Meatless Farm	
**PRODUCT DESCRIPTION**	Meat Free Burgers 2 x 113.5g	
**PRODUCT PACK SIZE**	227	
**PRODUCT EAN BARCODE**	5060626400412	
**Length mm**	125	
**Width mm**	219	
**Depth mm**	30	
**NET UNIT PACK WEIGHT (G)**<br>**Product content only**	227	
**GROSS UNIT PACK WEIGHT (G)**<br>**Includes product content + packaging**	249	
**UNITS PER CASE**	6	
**Length mm**	260	
**Width mm**	223	
**Depth mm**	108	
**NET CASE WEIGHT (G)**<br>**Product Content only**	1362	
**GROSS CASE WEIGHT (KG)**<br>**Includes product content + packaging**	1.66	
**CASE BARCODE**	15060626400419	
**PALLET TYPE**	EUR	GKN
**CASES ON PALLET LAYER (TI)**	15	18
**LAYERS ON PALLET (HI)**	15	13
**CASES ON PALLET (QTY)**	225	234
**UNIT PACKS ON PALLET (QTY)**	1350	1404
**NET PALLET WEIGHT (KG)**<br>**Product Content Only**	306.45	306.45
**GROSS PALLET WEIGHT (KG)**<br>**Includes product content + packaging and pallet weight**	585.25	610.66
**COMMODITY CODE**	21.06.102090	
**COUNTRY OF ORIGIN**	UK	
**TEMPERATURE BAND**	FROZEN	
**TOTAL PRODUCT LIFE DAYS**	365	

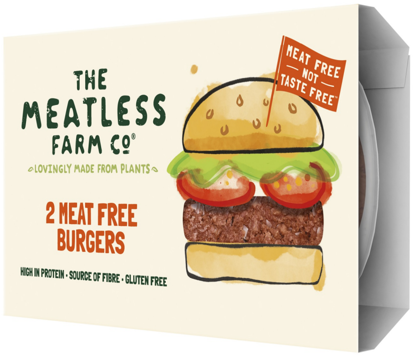

INGREDIENTS: Water, Pea Protein (19%), Rapeseed Oil, Shea Oil, Thickener: Methyl Cellulose, Coconut Oil, Rice Protein, Pea Fibre, Yeast Extracts, Caramelised Carrot Concentrate, Vegetable and Fruit Extracts (Beetroot, Radish, Tomato), Natural Flavourings, Potato Fibre, Potato Starch, Salt, Ground Black Pepper, Carrot Concentrate, Smoked Water, Acidity Regulator: Ascorbic Acid

ALLERGENS: **No allergens**

STORAGE: For Best Before, see top of pack. Keep frozen. Do not exceed use by date. Defrost thoroughly (in a refrigerator) before use. Once defrosted use within 24 hours. Do not re-freeze.

COOKING INSTRUCTIONS: Remove all packaging. Season both sides of your Meatless Burgers with salt and pepper or as desired. Heat a couple of tablespoons of oil in a non-stick pan on a medium heat. Add your burgers and cook for approximately 6-7 minutes turning occasionally. Ensure food is piping hot, serve and enjoy! Tip: Great on the BBQ!!

NUTRITIONAL INFORMATION PER 100G

KJ	957
Kcal	230
Fat	14.8g
of which saturated	4.7g
Carbohydrates	5.3g
of which sugars	0.3g
Fibre	3.7g
Protein	17.1g
Salt	1.49g

*Analytical results

SALES CONTACTS:
