# Supplementary material for: Impact of Plant-Based Meat Alternatives on the Gut Microbiota of Consumers: A Real-World Study
Source: Foods. 2021 Aug 30;10(9):2040. doi: 10.3390/foods10092040 (PMC8465665; doi:10.3390/foods10092040)
Supplement: Supplementary file 1 [file foods-10-02040-s001.zip › 6. Study Flow Diagram.pdf]

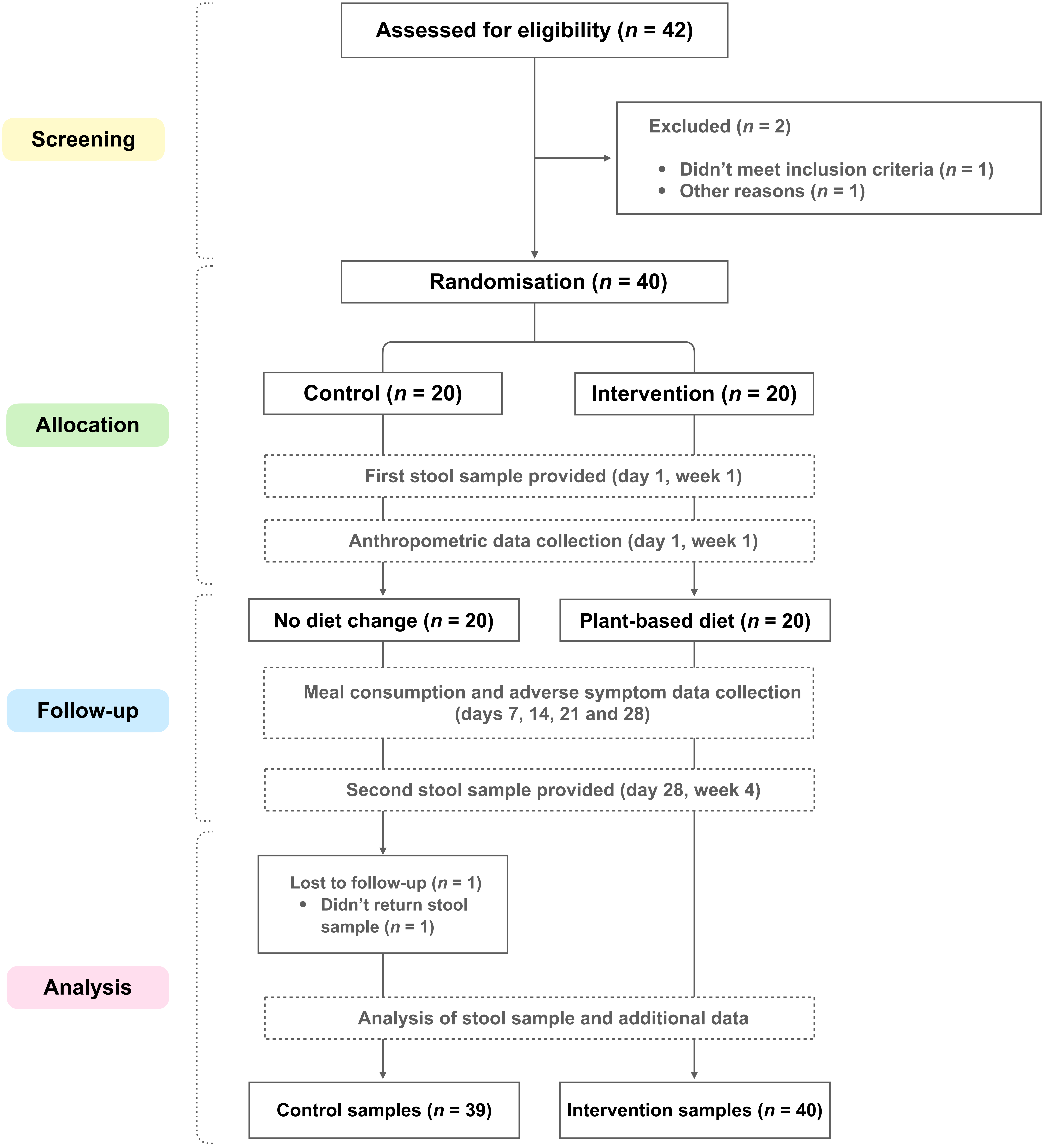
Screening
Allocation
Follow-up
Analysis
Assessed for eligibility (n = 42)
Excluded (n = 2)
• Didn't meet inclusion criteria (n = 1)
• Other reasons (n = 1)
Randomisation (n = 40)
Control (n = 20)
Intervention (n = 20)
First stool sample provided (day 1, week 1)
Anthropometric data collection (day 1, week 1)
No diet change (n = 20)
Plant-based diet (n = 20)
Meal consumption and adverse symptom data collection (days 7, 14, 21 and 28)
Second stool sample provided (day 28, week 4)
Lost to follow-up (n = 1)
• Didn't return stool sample (n = 1)
Analysis of stool sample and additional data
Control samples (n = 39)
Intervention samples (n = 40)
